# Visual Assessment of Methane Hydrate Dissociation Using a Multi-Rocking Cell: Roles of Surfactants, KHIs, and AAs in Water-Rich Systems

**DOI:** 10.1021/cbe.5c00067

**Published:** 2025-10-07

**Authors:** Sanehiro Muromachi, Michihiro Muraoka, Satoshi Takeya, Yoshihiro Konno, Kiyofumi Suzuki, Norio Tenma

**Affiliations:** a Graduate School of Engineering Science, 13154 Yokohama National University, 79-5 Tokiwadai, Hodogaya-ku, Yokohama 240-8501, Japan; b Energy Process Research Institute (EPRI), National Institute of Advanced Industrial and Science Technology (AIST), 16-1 Onogawa, Tsukuba 305-8569, Japan; c Department of Ocean Technology, Policy, and Environment, Graduate School of Frontier Sciences, The University of Tokyo, Kashiwa, Chiba 277-8561, Japan

**Keywords:** clathrate hydrate, subsea methane hydrate, inhibitor, fluidizer, rocking cell

## Abstract

This study uses a new multi-rocking cell system for visual, parallel assessment of methane hydrate (MH) dissociation. The method allows for systematic comparison of various fluidizers—composed of thermodynamic hydrate inhibitors like urea and additives including surfactants, kinetic hydrate inhibitors, and antiagglomerants—under controlled, water-rich conditions. Our findings show that surfactants, such as SDS, SO, and DDBSA, accelerate dissociation but cause MH migration and persistent foaming. In contrast, DTMAC and saponin effectively promote dissociation while suppressing foam and MH migration. Combination fluidizers (e.g., SDS + PVP, SDS + Tween 80) further fine-tuned the performance, demonstrating additive-specific effects such as foam suppression or accelerated dissociation. This work provides insights for designing effective fluidizers for hydrate management in subsea production environments.

## Graphical abstract

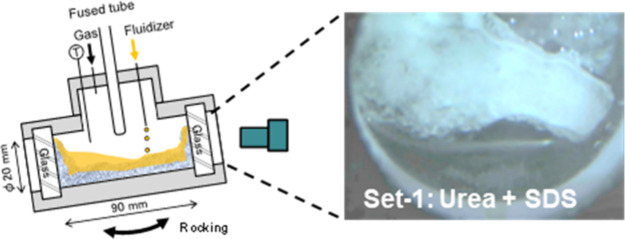

## 1 Introduction

Subsea methane hydrates (MH), which are predominantly found in subsea regions,^1^ have garnered significant attention due to their potential as a natural gas resource.^2,3^ Gas hydrates are solid crystals consisting of water and guest gases such as methane, carbon dioxide, and nitrogen, where the water molecules form cage-like cavities through hydrogen bonding, and large amounts of guest gases are encapsulated within the crystal.^4–7^ These gas hydrates are generally formed under low-temperature and high-pressure conditions, and in the case of MH, for example, they can exist stably under conditions of more than 2.7 MPa at 273 K. Efforts to develop effective gas production technologies from MH are crucial for tapping into these resources. While MHs in sandy-bed formations are considered an unconventional natural gas reservoir,^2,3^ those present in clayey sediments on the seafloor, known as shallow-type MH reservoirs, are also of increasing interest as a viable energy source.^8^ For instance, the sandy-bed type MH reservoirs generally exist under conditions of approximately 12 MPa pressure and 285 K temperature at the Nankai Trough in Japan.^1^ In contrast, shallow-type MH deposits, particularly those found in the waters surrounding Japan, typically form under seafloor temperatures of about 274 K,^9^ which are significantly lower than those of the sandy-bed reservoirs. Such low temperature conditions raise concerns about the formation of MH from methane gas and water released during MH dissociation, which may impede the production of methane gas. To date, as with oil and gas pipelines,^4,10^ formation of gas hydrates poses a serious risk of blockage and damage in pipelines and equipment because of their solid crystalline structure.^11^ Flow assurance technologies are essential to overcome these challenges and unlock the potential of MH resources.

Various inhibitors have been developed as countermeasures against hydrate-induced plugging in conventional oil and natural gas pipelines.^10^ These inhibitors can be broadly classified into the following three types: (i) Thermodynamic hydrate inhibitors (THIs): These inhibitors mainly include electrolytes such as NaCl and polar molecules such as methanol, which shift the MH formation conditions toward lower temperatures and higher pressures, thereby preventing MH formation. The inhibition effect of THI is based on the same principle as the freezing point depression of ice. They are effective when added at concentrations of 10 to several tens of mass% relative to water. (ii) Kinetic hydrate inhibitors (KHIs): These inhibitors are a class of low-dosage hydrate inhibitors (LDHIs) that include water-soluble amphiphilic polymers such as polyvinylpyrrolidone (PVP), which act on the crystal faces of hydrates and inhibit crystal growth and delay nucleation.^12^ The required concentration is on the order of several thousand parts per million (mass) relative to water. (iii) Antiagglomerants (AAs): These inhibitors, such as naphthenic acids, are also a class of LDHIs and hydrophobic substances that prevent the adhesion of hydrate crystals. Their required concentration is on the order of a few mass%. These three types of inhibitors have been extensively studied for approximately a century about antifreeze agents that are also used as THIs and for 30 years about KHIs and AAs,^12^ primarily in the context of conventional natural gas and oil pipelines, resulting in a wealth of accumulated knowledge. Applying these existing insights to subsea MH resource development can be a shortcut for developing gas recovery and plugging prevention technologies.

The applicability of existing inhibitors to subsea MH systems must be evaluated based on their unique characteristics. Conventional natural gas/oil pipelines operate at high pressures with hydrocarbons, such as ethane, making them highly susceptible to hydrate formation. In contrast, subsea MH wells contain primarily methane in a water-dominant environment with shorter transport distances and higher flow velocities, resulting in a lower tendency for hydrate formation. Based on these differences, hydrophobic AAs are unsuitable for water-dominant MH wells. The effectiveness of KHIs, which prevent crystal growth during long-distance transport, requires further study for short-distance, high-velocity conditions. THIs, however, are necessary to dissolve hydrate plugs that may reform downstream of the pump. Although THIs are used to decompose formed hydrates, their high required dosage presents a problem. In open-system subsea MH wells, the eventual discharge of THI-treated water requires environmental countermeasures like THI recovery or the use of environmentally benign THIs.^13–16^ Blockage from MH reformation remains a risk during all production and separation processes, particularly under shut-in conditions. This risk is amplified in cold regions such as the Sea of Japan, where low seafloor temperatures (∼274 K) can affect even surface facilities. Consequently, the development of technologies using inhibitors to ensure hydrate flowability is essential to mitigate plugging risks.

In this study, we report visualized laboratory tests of MH fluidizers composed of THIs, KHIs, and AAs. For systematic evaluation, we developed a custom multi-rocking cell apparatus, a method well-established for reproducible hydrate research.^17–20^ This system enables direct visual observation of hydrate dissociation and fluidization behavior, facilitating the comparison of different fluidizer compositions. In our evaluations, urea was used as the THI in all formulations due to its effective performance and environmentally friendly properties compared to conventional THIs such as methanol and ethylene glycol.^13,14^ Supporting additives included surfactants such as sodium dodecyl sulfate (SDS), dodecyl, trimethylammonium chloride (DTMAC), sodium oleate (SO), dodecylbenzenesulfonic acid (DDBSA), polyoxyethylene sorbitan monooleate (Tween 80), lauryl dimethylaminoacetic acid (LDAAA) and saponin, and polyvinylpyrrolidone (PVP) as the KHI were used with urea. These additives were selected based on their diverse properties, including commercial availability, biodegradability, and expected roles in reducing flow resistance, promoting mixing, inhibiting hydrate formation or agglomeration, and improving dissociation behavior. This comprehensive comparison helps clarify the distinct mechanisms of these additives and their applicability to subsea MH dissociation in water-rich systems.

## 2 Experimental Section

Detailed procedures are provided in the Supporting Information. Here, we provide a brief explanation. We used the raw materials listed in Table 1, which also provides the properties of surfactants, i.e., Krafft point and critical micelle concentration (CMC), used in this study. Based on the literature data, the Krafft points of SDS, SO, and DDBSA are beyond the test temperature, i.e., 10 °C, which suggests that these surfactants do not form micelles under the present test conditions. In this study, urea was employed as the THI component in all fluidizer formulations. The concentration of this aqueous solution was set at 30 mass% for all fluidizers. While surfactants are expected to promote hydrate dissociation by reducing flow resistance and improving mixing, they can also paradoxically promote hydrate formation.^21–27^ The effect of a common surfactant, SDS, for instance, is highly dependent on its concentration.^22^ Conversely, other surfactants such as the cationic DTMAC and SO have been reported to have no promoting effect on MH formation.^23^ This suggests that with these compounds, only the positive effects of reducing flow resistance and enhancing mixing can be expected. PVP, used as a KHI, is known to inhibit hydrate crystal growth at concentrations below 1 mass%. By combination of KHI with surfactants, it is also expected that the mutual benefits of these agents can facilitate rapid fluidization. DDBSA, which is expected both to promote hydrate formation^23^ and to reduce hydrate cohesive force,^28^ is used as a component of a fluidizer. LDAAA is an amphoteric surfactant. In addition to LDAAA, we also used saponin, which is made from soybeans and thus expected to be biodegradable. Tween 80, which is a nonionic surfactant also used as an AA,^29^ was also evaluated.

**Table 1:** List of Materials Used in This Study^a^

name	chemical formula	CAS Registry Number	supplier	purity	Krafft point/°C	CMC/ppm^b^
methane	CH_4_	74–82–8	Tokyo Gas Chemicals Co., Ltd.	≥99.995 vol %		
urea	CH_4_N_2_O	57–13–6	Fujifilm Wako Chemical Co., Ltd.	≥0.999 in mass fraction		
sodium dodecyl sulfate (SDS)	NaC_12_H_25_SO_4_	151–21–3	Junsei Chemical Co., Ltd.	>0.99	16 ^30^	2600 at 5 °C^31^
polyvinylpyrrolidone (PVP)	(C_6_H_9_NO)_n_	9003–39–8	Tokyo Chemical Industry Co., Ltd.			
sodium oleate (SO)	C_17_H_33_COONa	143–19–1	Tokyo Chemical Industry Co., Ltd.	>97.0%	27 ^32^	900 at 25 °C^33^
dodecyltrimethylammonium chloride (DTMAC)	C_15_H_34_ClN	112–00–5	Tokyo Chemical Industry Co., Ltd.	>98.0%	<0 ^34^	4200 at 25 °C^35^
polyoxyethylene sorbitan monooleate (Tween 80)	C_64_H_124_O_26_	9005–65–6	Tokyo Chemical Industry Co., Ltd.			20 at 25 °C^36^
dodecylbenzenesulfonic acid (DDBSA)	C_18_H_29_SO_3_Na	68584–22–5	Tokyo Chemical Industry Co., Ltd.	>95.0%	52 ^37^	450 at 25 °C^38^
lauryl dimethylaminoacetic acid (LDAAA) 35% solution	C_16_H_33_NO_2_	683–10–3	Fujifilm Wako Chemical Co., Ltd.		<1 ^39^	500 at 25 °C^40^
saponin (soyasaponin)	C_48_H_78_O_18_	8047–15–2	Fujifilm Wako Chemical Co., Ltd.			100 at 25 °C^41^

a For surfactants, the Krafft point and CMC at atmospheric pressure conditions are also provided.

b Mass basis ppm estimated from the reference data.

A schematic diagram of the newly designed and constructed rocking cell flow experiment apparatus is shown in Figure 1. The test section inside the rocking cell is a cylindrical chamber with an inner diameter of 20 mm and a length of 89 mm. It has an optical observation window along its longitudinal direction, enabling direct observation via the naked eye or a camera. The cylindrical test section had an internal volume of approximately 28 cm^3^. A fused tube was equipped with each cell from approximately 20 mm above the bottom of the cell, near its center, where a cold rod was inserted for facilitating nucleation of MH. The rocking angle and period were 15° and 8 s, which were primarily determined by the mechanical limitations of the laboratory apparatus. However, these parameters can also be regarded as a reasonable representation of the limited agitation that would be available in actual subsea MH production systems, where hydrate blockage occurs and only minor motions caused by equipment vibrations or ocean currents can be expected.

**Figure 1:**
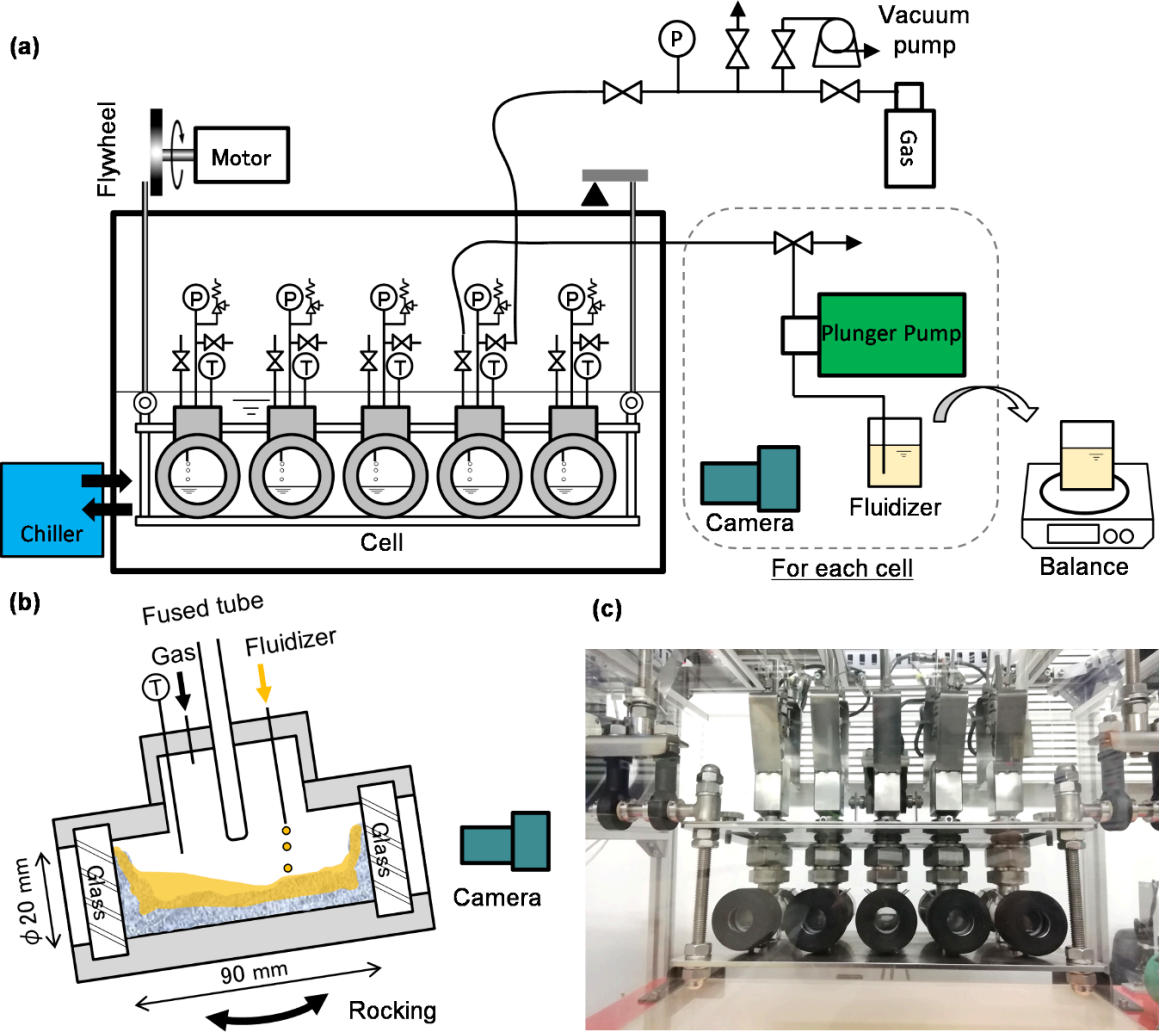
Multi-rocking cell device for the MH dissociation test. (a) A schematic diagram of the whole system of the device. (b) Dimension of the test cell (not to scale) and conceptual explanation for fluidizer injection. (c) A picture of the cells set on the rocking device.

### Procedures

Approximately 5 g of water was introduced into the rocking cell. MH was formed at 10 MPa and 283.2 K. A cooling rod was inserted into the fused tube equipped with the cell to locally cool the internal environment, which promoted hydrate nucleation. The cell was rocked continuously until the internal fluid stopped flowing, indicating complete hydrate formation. Approximately 1 g of the fluidizer was injected into the rocking cell using a plunger pump from Cell No. 1 to No. 5. The mass of the injected fluidizer was determined by measuring the mass of the fluidizer bottle before and after injection. The flow state was documented by recording a video at 10 min intervals. The waiting time between successive injections varied between 30 min and 2 h, depending on the degree of observable change. These procedures were repeated until complete hydrate dissociation was achieved.

In our experiments, the injection interval was not predetermined but adjusted so that after each injection, the system was allowed to evolve until no further observable changes occurred. This approach was taken in order to capture the dissociation and morphological state as close as possible to a quasi-steady condition for each injection step. This design also reflects practical considerations: in actual gas production systems for subsea MHs, the degree of mixing and the time scale of blockage development cannot be precisely predicted. In severe plugging events, the only countermeasure would be to inject a large amount of strong THI. In less severe cases, however, it would be more desirable to allow sufficient time for the injected inhibitor to interact with the hydrate, thereby minimizing the environmental impact by reducing the total amount of chemical injection. For these reasons, the experimental protocol was deliberately designed to vary the waiting time between injections, depending on the observed system response.

Full descriptions of the experimental parameters used are given in Table S1 in the Supporting Information. Table 2 summarizes the composition of the fluidizers used in this experiment. These conditions are denoted as “Set” in the table. In this study, the effects of surfactants at different concentrations were examined by injecting the fluidizers and observing the hydrate formation and dissociation behavior.

**Table 2:** Nominal Parameters of MH Dissociation Tests Performed in This Study^a^

				parameter (nominal)				
run name	THI	Additive 1 (Variant)	Additive 2	No. 1	No. 2	No. 3	No. 4	No. 5
Set-1		SDS		0	50 ppm	500 ppm	1000 ppm	5000 ppm
Set-2		PVP		0	1%	2%	5%	10%
Set-3		SO		0	50 ppm	500 ppm	1000 ppm	5000 ppm
Set-4		DTMAC		0	50 ppm	500 ppm	1000 ppm	5000 ppm
Set-5	urea (30%)	Tween 80		0	100 ppm	500 ppm	1000 ppm	5000 ppm
Set-6		DDBSA		0	50 ppm	500 ppm	1000 ppm	5000 ppm
Set-7		LDAAA		0	50 ppm	500 ppm	1000 ppm	5000 ppm
Set-8		saponin		0	50 ppm	500 ppm	1000 ppm	5000 ppm
Set-9		PVP	SDS (1000 ppm)	0	1%	2%	5%	10%
Set-10		Tween 80	SDS (1000 ppm)	0	100 ppm	500 ppm	1000 ppm	5000 ppm

a Detail parameters are provided in Table S1 in the Supporting Information. Compositions of the components are mass basis and shown as that in the binary mixture of water and the component.

Figure 2 shows the present conditions for the MH dissociation tests in the phase diagram. The present pressure and temperature conditions, i.e., 10 MPa and 283.2 K, are on the phase equilibrium curve of MH inhibited by 10 mass% urea solution. Table 3 shows the simulated concentration of the fluidizer components in the aqueous solution in the cells on the assumption of ideal complete mixing of the fluidizer and the initially injected water. Based on this calculation, at the third injection, the urea concentration reaches over 10 mass% urea. Therefore, after infinite waiting time, MH in the cell may completely dissociate. If not in this case, it means that the mixing in the cell is not enough, and MH is kinetically preserved in the system, which does not reach an equilibrium state.

**Figure 2:**
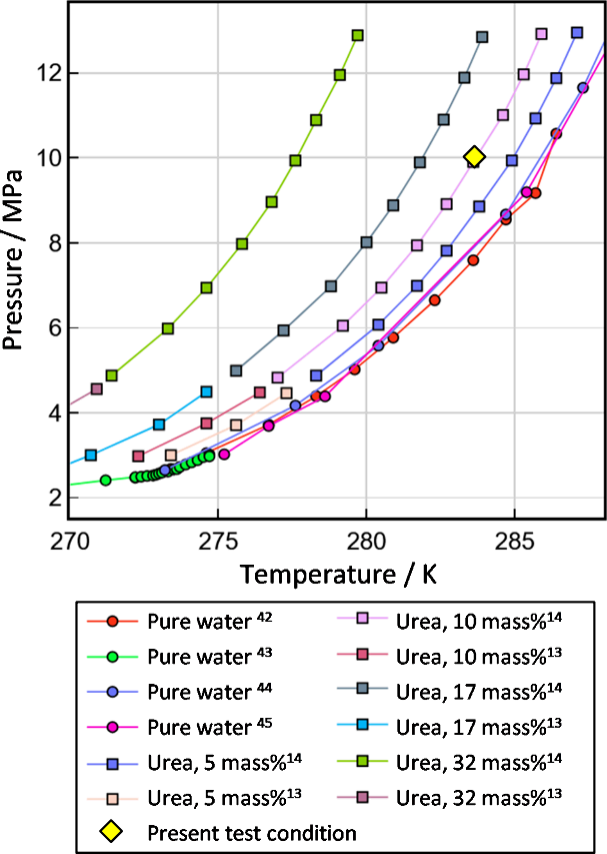
Present test conditions are shown on the phase diagram of MHs. The phase equilibrium data for the pure water system and the systems inhibited by urea are taken elsewhere.^13,14,42–45^

**Table 3:** Simulated Concentration of the Components through Fluidizer Injection under Ideal Complete Mixing Conditions

			concentrations under ideal complete mixing conditions				
	fluidizer			surfactant			
initial water (g)	injection	g	urea mass fraction	No. 2 (100 ppm)	No. 3 (500 ppm)	No. 4 (1000 ppm)	No. 5 (5000 ppm)
5	0th	0	0.00	0	0	0	0
	1st	1	0.05	18	88	175	877
	2nd	2	0.09	31	156	313	1563
	3rd	3	0.11	42	211	423	2113
	4th	4	0.13	51	256	513	2564
	5th	5	0.15	59	294	588	2941
	6th	6	0.16	65	326	652	3261
	7th	7	0.18	71	354	707	3535
	8th	8	0.18	75	377	755	3774

## 3 Results and Discussion

The full sequential pictures and the pressure and temperature trends during each Set are provided in Figures S1 and S2, respectively, in the Supporting Information. As shown in the pressure–temperature trends, no distinct pressure change attributable to hydrate formation was observed. This is considered to result from the relatively small amount of water (5 g) and the modest mixing intensity in the cell. Nevertheless, the focus of this study is on the fluidization behavior of MH. Under the selected conditions (10 MPa and 10 °C), MH blocks were successfully formed and became completely immobile prior to fluidizer injection, ensuring that the intended experimental setup was achieved. The Supporting Information also provides the full video of MH dissociation. Figure 3 shows a typical dissociation behavior of MH selected from Set-1. Before MH formation, water species in the cells were moved by the rocking stage. In this figure, MH eventually formed in Cell No. 5; however, such heterogeneous nucleation does not affect the subsequent MH dissociation tests because the morphology of the formed MH is the same in the five cells regardless of their nucleation behaviors. After MH formation (0th image in the figure), the MH sticks to the glass window. Subsequently, about 1 g of fluidizer is injected into each cell. The fluidizer injection caused a visual change in the cell as shown in this figure. At sixth to eighth injection, the MH in all the cells had dissociated. In all of the Sets, after the third fluidizer injection, which may completely dissociate MH under ideal complete mixing conditions, MH persistently remained in the cells. This fact suggests that the MH block formed in the cells slowly dissociates and the additives are required for the rapid MH dissociation. The following sections discuss the effects of different fluidizer compositions and comparative conditions.

**Figure 3:**
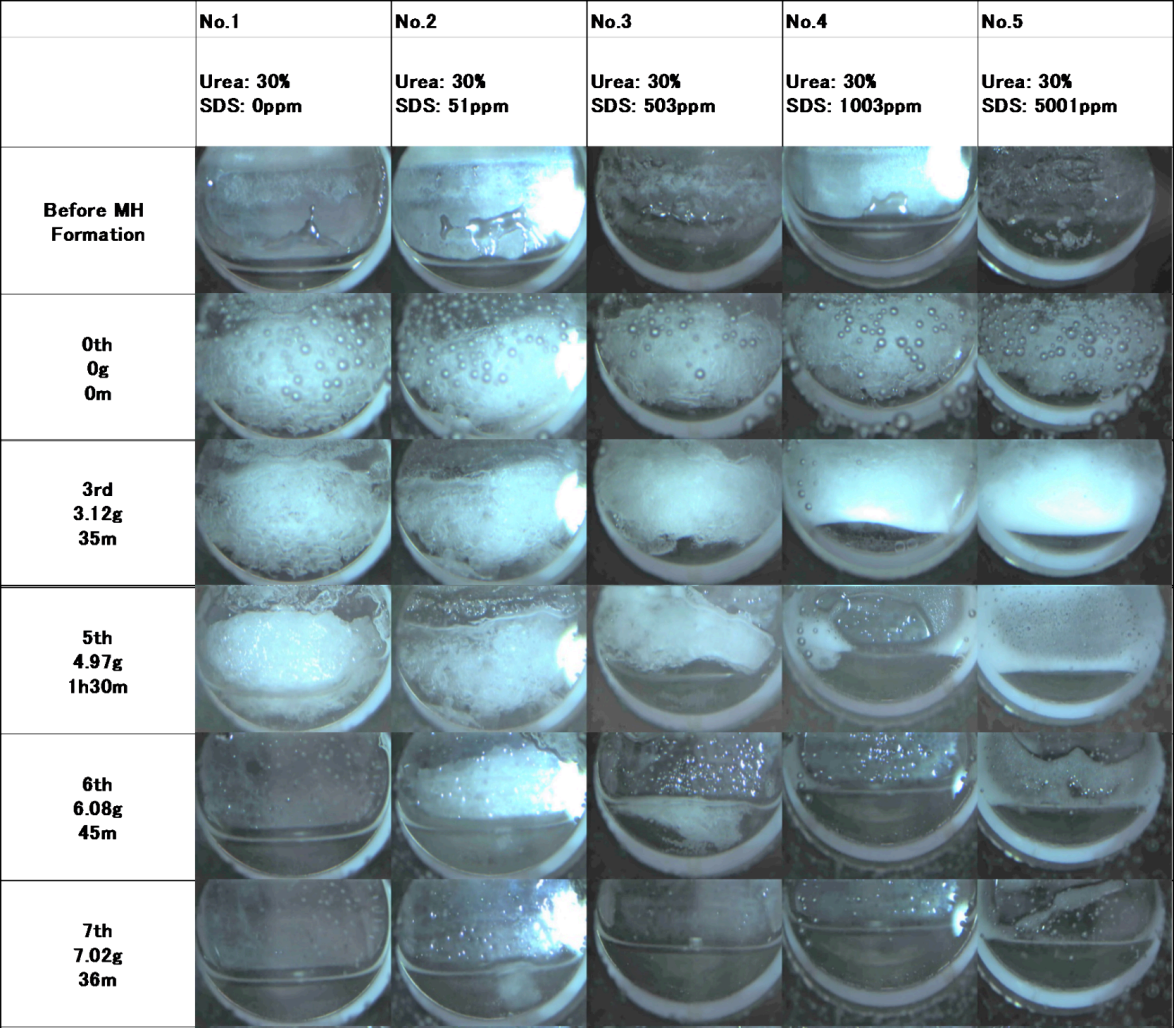
Typical dissociation behavior of MH. Selected pictures are from Set-1. The window of Cell No. 2 reflects the light source at the right side of the image. In the zeroth image, air bubbles are on the outside of the glass window.

### Set-1: SDS

As shown in Figure 3 and Figure S1a, the foamability of SDS affects MH dissociation. The MH dissociation effect was confirmed for five different SDS concentrations of the fluidizer. Although the fluidizer is diluted after injection into the MH-producing system at the early stage, migration of MH toward the top of the cell was immediately observed (see first and second injections in Figure S1a). Here, “migration” describes the reformation process of MH inside the rocking cell, characterized by the detachment of hydrate from the bottom surface and its subsequent upward movement within the cell. At the third injection, the window of Cell Nos. 3–5 was covered with the foam and MH, which are hard to distinguish optically. It is suggested that the SDS concentration in the fluidized part may exceed the CMC, i.e., 2600 ppm. At the bottom of these cells, moving liquid is observed, whereas it was not observed in Cell Nos. 1 and 2, which suggests that the fluidizer containing SDS can remove MH from the bottom. Subsequent injections reduced the MH in the cells. At the fifth injection, most of the MH in Cell Nos. 3–5 was dissociated and the cells were fluidized. In contrast to these cells, the MH in Cell Nos. 1 and 2 where the fluidizers containing no or a small amount of SDS were used still remained at the same stage. This suggests that dissociation or removal of MH can be facilitated by doping the surfactant into THI with an appropriate amount. Following the sixth injection, complete MH dissociation led to thorough mixing of the components, reducing the SDS concentration below the critical micelle concentration (CMC) in all cells except Cell No. 5, where foaming remained observable.

### Set-2: PVP (See Figure S1b)

In Set-2, experiments were conducted using PVP as the sole supporting component for THI. Since PVP is also used as a water absorbent, the adjusted fluidizers are viscous liquids. As shown in Figure S1b, no significant promotion of MH dissociation was observed. On the contrary, Cell No. 1 dissociated earlier than the others, which suggests that PVP may have slower dissociation of MH due to the increased viscosity at high PVP concentrations with 0–10 mass%. Another possibility is the dissociation retarding effect of KHI.^46,47^ This effect reported that the MH dissociation rate becomes lower by an order of magnitude than that usual with KHIs. However, based on our observations, we believe that the high viscosity likely hindered the contact between the THI and the MH. PVP is generally effective at concentrations on the order of 0.1 mass%,^4,12^ and thus, the currently doped amounts are excessive. However, considering the dilution of PVP in the injected system, a dense solution such as used in this study may be preferred.

### Set-3: SO (See Figure S1c)

We used SO with the same fluidizer compositions with Set-1, i.e., SDS. A quite similar dissociation behavior to Set-1 was observed in each cell. The MH at the bottom of the cells dissociated as fluidizer injection instead of migration of the MH to the top part of the cell. In Cell Nos. 2 and 3, MH possibly dissociated earlier than Set-1 at the fourth or fifth injection. This means that SO may facilitate MH dissociation with a smaller amount than SDS. At the last stages, the sixth and seventh injection, fine bubbles remained in the Cell Nos. 3–5, although SDS did not keep foaming in Set-1. SO may effectively work with 50 ppm as a supporting agent for THI, urea in this case. We note that SO generated foam in these systems, while the present test temperature, 10 °C, is below the Krafft point of SO at 27 °C.

### Set-4: DTMAC (See Figure S1d)

Compared to SDS, MH decomposition was faster when using DTMAC. In contrast, in the experiment using DTMAC (Set-4), MH dissociation was completed in cells 4 and 5 at the fifth injection stage, while MH was not fully dissociated in the SDS experiment. Notably, no foam generation was observed when using DTMAC even with high concentrations, i.e., 1000 and 5000 ppm, while prominent foam generation was consistently observed in the experiments using SDS and SO. Furthermore, in the case of DTMAC, no migration of MH within the cell was detected. These findings indicate that DTMAC not only suppresses foam generation and MH migration but also effectively promotes MH dissociation. Such foaming behavior was plausible based on the Krafft point (<0 °C) and the CMC (4200 ppm) of DTMAC, both which are out of requirements for forming a micelle.

### Set-5: Tween 80 (See Figure S1e)

In Set 5, where Tween 80 was used, migration of MH within the cell was clearly observed. Although Tween 80 was employed as an AA rather than a surfactant, it exhibited migration behavior similar to that induced by SDS. Notably, MH migration was also observed in the system containing approximately 100 ppm Tween 80, highlighting its significant influence even at low concentrations. However, the overall effect of Tween 80 on MH fluidization appeared to be lower than that of conventional surfactants such as SDS, as a considerable amount of MH remained in the cells even after the fifth injection of the fluidizer.

Based on this experimental result, although Tween 80 has been reported to function effectively as an AA in oil- and gas-rich pipeline systems, its performance appears to be limited in water-rich environments such as the present subsea MH systems. Specifically, Tween 80 exhibited little to no observable effect in MH fluidizing under these conditions, suggesting that it may not be suitable as an effective component of an MH fluidizer for such applications.

### Set-6: DDBSA (See Figure S1f)

In the experiment of Set-6 using DDBSA, the MH dissociation pattern was found to closely resemble that observed in Set 3, where the SO was used. Specifically, as the fluidizer was incrementally injected, MH migrated upward within the cell while dissociation progressed from the bottom. In addition, pronounced foam formation was observed, and in Cell Nos. 3 to 5, the foam persisted even after the MH had dissociated, although the Krafft point of DDBSA was 52 °C.

### Set-7: LDAAA (See Figure S1g)

In Set-7, where LDAA was used, no clear promotion of MH dissociation was observed, unlike in the case with SDS. However, in Cell No. 5, the foam generation behavior was similar to that observed with SDS or SO, which persisted even after the MH had dissociated. The foam generation is only observed in Cell No. 5 in compliance with CMC.

### Set-8: Saponin (See Figure S1h)

In Set-8, where saponin, a surfactant derived from soybeans, was used as a surfactant, MH dissociation was promoted to an extent similar to that observed with SDS and DTMAC. The key differences, however, were related to foam generation and MH migration within the cells. While MH migration was not observed with DTMAC but was prominent with SDS, Set-8 showed moderate migration behavior: MH migration was observed in Cell Nos. 3–5, though less pronounced than in the SDS case. Notably, despite the presence of MH migration, no significant foam formation was observed throughout the process—from the beginning of flow agent injection until complete MH dissociation.

### Comparison between Surfactants, KHI, and AA

Figure 4 compares Set-1 to Set-8 for Cell No. 3 at the third and fifth injection stages. In Set-1 (SDS), Set-3 (SO), Set-6 (DDBSA), and Set-8 (saponin), clear MH dissociation at the bottom of the cell was observed at the third injection. This is due to the promotion effect of these surfactants on the MH migration. In the other sets, any clear MH dissociation in Cell No. 4 was not observed. At the fifth injection stage, a large MH block remained in Set-1 (SDS), Set-2 (PVP), and Set-5 (Tween 80). Since the fluidizer with 1000 ppm SDS (Cell No. 4 in Set-1) dissociated MH, 500 ppm SDS may be insufficient in this test. PVP and Tween 80 gradually shaved the initially formed MH block from its surface, but the MH block remained at this stage. In Set-4 (DTMAC) and Set-7 (LDAAA), the small MH block also remained (for Set-4, MH block appeared at the sixth injection, see Figure S1d), which suggests that these additives slightly promoted the MH dissociation compared to PVP and Tween 80. Set-3 (SO), Set-6 (DDBSA), and Set-8 (saponin) dissociated MH by the fifth injection. Although the prominent foam is observed with SO and DDBSA, DTMAC and saponin did not generate foam clearly. This comparison proposed that the additives, i.e., surfactant, KHI, and AA, can change the MH dissociation behavior. For immediate removal of MH from where it formed, surfactants that generate foam is suitable as additives, SDS, SO, and DDBSA, in this study. LDAAA may provide a mild MH removal effect. DTMAC and saponin work for promoting MH dissociation with suppression of the MH migration. Some tested surfactants, i.e., SDS, SO, and DDBSA, generated foam below their Krafft points, which may be induced by MH dissociation.

**Figure 4:**
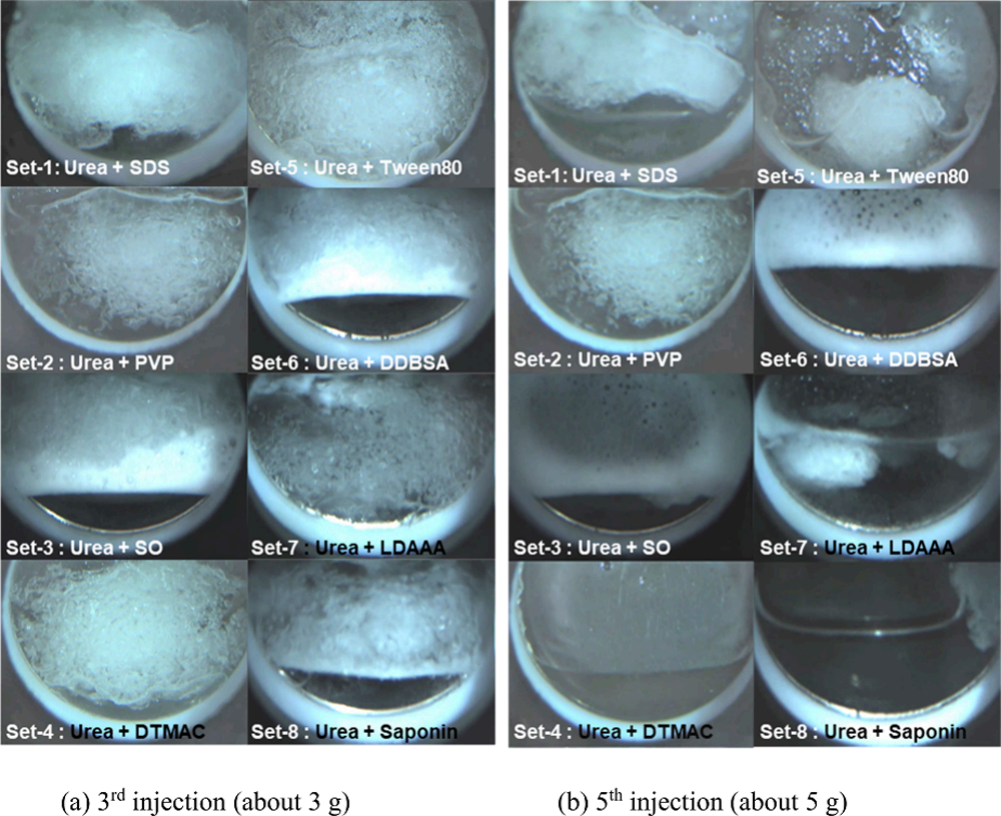
Comparison between Set-1 to Set-8 for Cell No. 3 at the 3rd and 5th injection stages.

### Combination of Surfactant and KHI or AA: Set-9 and Set-10 (See Figure S1i and S1j)

In fluidizers for Set-9, SDS was added at 1000 ppm, and the effect of different PVP concentrations was examined. When the PVP concentration in the fluidizer was below 1 mass%, the suppression of MH reformation at the top of the cell by SDS was insufficient. When the PVP concentration was 5 mass%, MH dissociation was observed upon the fifth injection, whereas at other concentrations, dissociation occurred at a later stage. In Cell No. 5, MH did not change clearly until the sixth injection, and it suddenly dissociated after the seventh injection. This is likely due to the dense PVP, which increased the viscosity of the fluidizer. These results indicate that there is an optimal PVP concentration in the fluidizer, which was found to be 5 mass% in this experimental system. In Set-10, experiments were conducted using a fluidizer containing both Tween80 and SDS. In the presence of 1000 ppm Tween 80, MH dissociation was nearly complete upon the fourth injection. In Set-9, MH in Cell No. 5 remained even after the fifth injection, which suggests that the dense Tween 80 may delay MH dissociation. Comparatively, in Set-9 (SDS + PVP) and Set-4 (DTMAC), MH dissociation was achieved upon the fifth injection, indicating that MH dissociation occurred earlier in Set-10 (SDS + Tween 80).

Figure 5 clearly shows the effects of the double additive fluidizers. The urea solution without an additive (Cell No. 1) needs more injection than the fluidizers with additives. With SDS as a single additive (Set-1), MH at the bottom of the cell was already removed. The double additive SDS + Tween 80 (Set-10) shows similar behavior. PVP suppresses foam generation compared to Set-1 (SDS) and Set-10 (SDS + Tween 80). After the fifth injection, all the double additive systems dissociated MH. A difference can be found in the foam generation: no foam in Set-9 (SDS + PVP) and with foam in Set-1 (SDS) and Set-10 (SDS + Tween 80).

**Figure 5:**
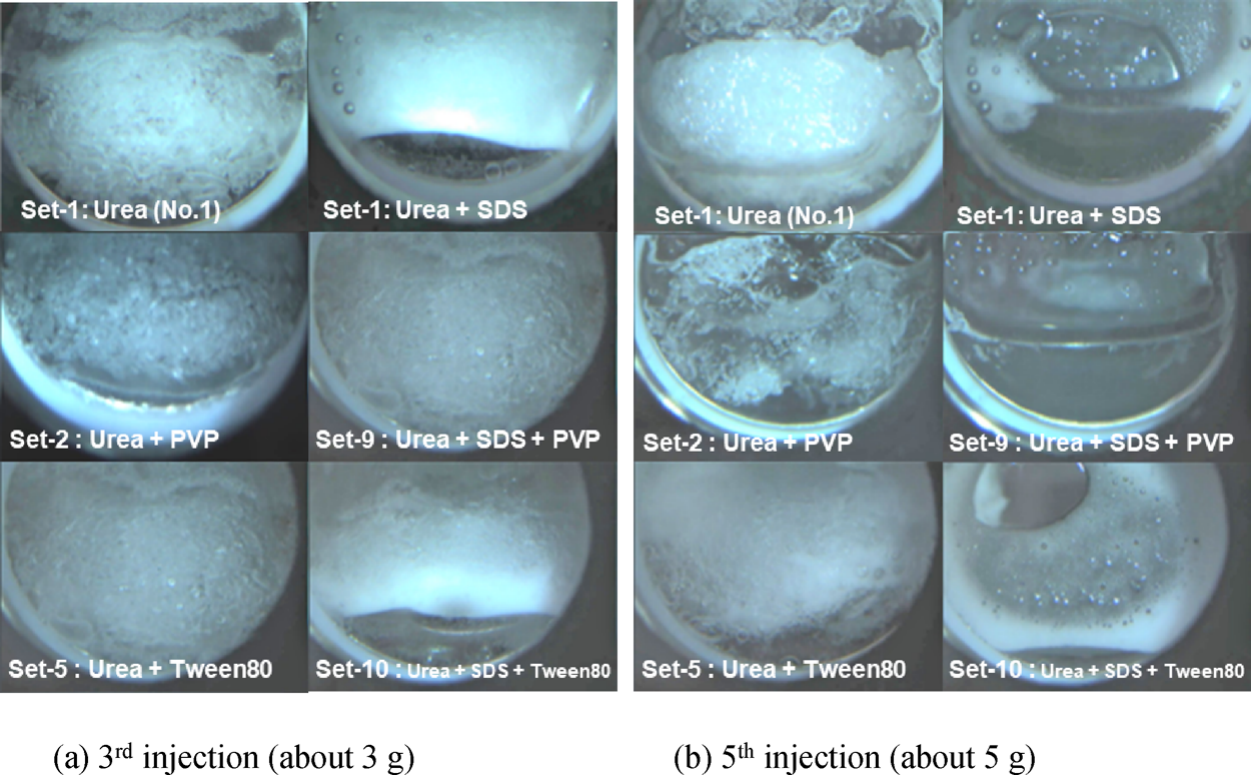
Comparison between single and double additive fluidizers for Cell No. 4 at the 3rd and 5th injection stages. The SDS concentrations used in these sets are the same, i.e., 1000 ppm.

## 5 Conclusions

In this study, we successfully utilized a multi-rocking cell device to systematically evaluate the dissociation behavior of MH in the presence of fluidizers composed of urea-based THIs and various additives, including surfactants, KHIs, and AAs. Table 4 summarizes the present experimental results. The distinct roles of each additive in MH dissociation are linked to their molecular properties. While anionic surfactants lower interfacial tension and promote dissociation, they can also stabilize foam, a problem mitigated by cationic and nonionic surfactants. Conversely, kinetic hydrate inhibitors and antiagglomerants showed limited dissociation efficacy, as their primary functions are to inhibit growth and prevent particle aggregation, respectively. Overall, the results indicate that selecting appropriate surfactant and additive combinations allows for control over not only the rate of MH dissociation but also undesirable phenomena such as foaming and MH migration. The insights from this work provide a practical basis for designing effective fluidizer systems tailored to subsea hydrate production, particularly in water-rich shallow reservoirs where hydrate reformation poses a critical operational challenge.

**Table 4:** Summary of the Experimental Results

set	additive	foaming	MH migration	dissociation
1	SDS	yes (persistent)	yes	complete, slower in low conc. cells
2	PVP	no	no	slower due to viscosity; incomplete at high conc.
3	SO	yes	yes	complete, with residual fine bubbles
4	DTMAC	no	no	complete, faster than SDS; stable
5	Tween 80	limited	yes	partial; less effective than SDS
6	DDBSA	yes	yes	complete, foam persisted
7	LDAAA	yes (at high conc.)	mild	limited promotion, incomplete
8	saponin	no	moderate	complete, without foam
9	SDS + PVP	no	limited	complete, foam suppressed
10	SDS + Tween 80	yes	yes	complete, with foam

## Supplementary Material





## Notes

Full descriptions of experimental parameters used (Table S1). The full sequential pictures and the pressure and temperature trends during experiments (Figures S1 and S2) (PDF)
The full video of MH dissociation (MP4)
