# Supplementary material for: Visual Assessment of Methane Hydrate Dissociation Using a Multi-Rocking Cell: Roles of Surfactants, KHIs, and AAs in Water-Rich Systems
Source: Chem Bio Eng. 2025 Oct 7;3(1):28–36. doi: 10.1021/cbe.5c00067 (PMC12833707; doi:10.1021/cbe.5c00067)
Supplement: Supplementary file 1 [file be5c00067_si_001.pdf]

Supplementary Information for:

Visual Assessment of Methane Hydrate Dissociation Using a Multi-Rocking Cell: Roles of Surfactants, KHIs, and AAs in Water-Rich Systems

Sanehiro Muromachi[a,b*], Michihiro Muraoka[b], Satoshi Takeya[b], Yoshihiro Konno[c], Kiyofumi Suzuki[b], Norio Tenma[b]

[a] *Graduate School of Engineering Science, Yokohama National University, 79-5 Tokiwadai, Hodogaya-ku, Yokohama 240-8501, Japan*

[b] *Energy Process Research Institute （EPRI）, National Institute of Advanced Industrial and Science Technology (AIST), 16-1 Onogawa, Tsukuba 305-8569, Japan*

[c] *Department of Ocean Technology, Policy, and Environment, Graduate School of Frontier Sciences, The University of Tokyo, Kashiwa 277-8561 Chiba, Japan*

* Author to whom correspondence should be addressed.

S.M.

Tel : +81-45-339-4009

E-mail : muromachi-sanehiro-sf@ynu.ac.jp

Experimental

Materials

We used the raw materials listed in Table 1. Deionized water was filtered through an activated carbon filter and subsequently sterilized using ultraviolet (UV) light before use. Sample preparation was conducted on a mass basis using an analytical balance. This table also provides properties of surfactants, i.e., Krafft point and critical micelle concentration (CMC), used in this study. Based on the literature data, Krafft points of SDS, SO and DDBSA are beyond the test temperature, i.e., 10 °C, which suggests these surfactants do not form micelles under the present test conditions.

In this study, urea was employed as the THI component in all fluidizer formulations. Since urea is a solid under normal conditions, it was mixed with water to form an aqueous solution before being injected into the experimental system, and the concentration of this aqueous solution was set at 30 mass% for all fluidizers. In terms of fluidization, surfactants are expected to promote hydrate dissociation by reducing flow resistance and facilitating the mixing of the hydrate and inhibitor, although they often promote hydrate formation by capillary effects[21–27]. SDS, one of the most widely-used surfactant, is known to promote hydrate formation under certain conditions. According to Okutani et al.[22], the rate of hydrate formation increases as the SDS concentration rises from 0 ppm and saturates at approximately 1000 ppm, while slightly decreasing at around 4000 ppm. Furthermore, at low concentrations, no promotion effect was observed at 80 ppm, whereas it was confirmed at 125 ppm at a stationary condition. This indicates that the effect of surfactants on hydrate formation varies depending on concentration. On the other hand, DTMAC , which is a cationic surfactant, and SO have been reported to have no promoting effect on MH formation[23], suggesting that only the effect of reducing flow resistance and promoting mixing can be expected. PVP, used as a KHI, is known to inhibit hydrate crystal growth at concentrations below 1 mass%. In MH production technology, it is necessary to suppress MH reformation, making the use of KHI as a fluidizer promising. By combining KHI with surfactants, it is also expected that the mutual benefits of these agents can facilitate rapid fluidization. DDBSA, which is expected both to promote hydrate formation[23] and to reduce hydrate cohesive force[28], is used as a component of fluidizer. LDAAA is an amphoteric surfactant. In addition to LDAAA, we also used saponin which is made from soybeans and thus expected to be biodegradable. Tween 80, which is a nonionic surfactant also used as an AA[29], was also evaluated.

Apparatus

A schematic diagram of the newly designed and constructed rocking cell flow experiment apparatus is shown in Fig. 1. The apparatus mainly consists of five identical rocking cells, a rocking motor, a thermostatic bath, a plunger pump for inhibitor injection, and a camera for in situ observation of the cell interior. The cells were made of stainless steel. The test section inside the rocking cell is a cylindrical chamber with an inner diameter of 20 mm and a length of 89 mm. It has an optical observation window along its longitudinal direction, enabling direct observation via the naked eye or a camera. The cylindrical test section has an internal volume of approximately 28 cm^3. A fused tube was equipped with each cell from approximately 20-millimeters above the bottom of the cell, near its center, where cold rod was inserted for facilitating nucleation of MH. The pressure of the cell was measured by a pressure sensor (Keyence, GP-M100). Temperatures of the inside of the cells were measured by platinum resistance thermometer (Ichimura-metal, Pt100Ω). The rocking motion causes the cell to oscillate back and forth, inducing fluid impact against the observation window along the longitudinal axis of the cylinder. The rocking angle and period were 15° and 8 seconds, which were primarily determined by the mechanical limitations of the laboratory apparatus. However, these parameters can also be regarded as a reasonable representation of the limited agitation that would be available in actual subsea methane hydrate production systems, where hydrate blockage occurs and only minor motions caused by equipment vibrations or ocean currents can be expected.

The injection of a fluidizer increases the internal pressure of the cell; however, an overpressure relief valve installed in the gas piping releases gas to the external environment when the preset pressure is exceeded, thereby maintaining a constant internal pressure. In this study, methane hydrate (MH) was generated in five rocking cells. Afterward, fluidizers with inhibitors and additives at varying concentrations

were introduced to induce MH dissociation, enabling the simultaneous acquisition of dissociation behavior data under different experimental conditions.

Procedures

The experimental procedure consists of two major stages: (i) MH formation and (ii) MH dissociation. The detailed procedures for each stage are described below.

(i) MH Formation

1. Approximately 5 g of water was introduced into the rocking cell.
2. The cell was sealed, evacuated, and subsequently filled with methane gas up to 10 MPa. The temperature was then lowered to 283.2 K, the designated test temperature for hydrate formation and dissociation, and held until thermal equilibrium was reached.
3. A cooling rod was inserted into the fused tube equipped with the cell to locally cool the internal environment, promoting hydrate nucleation.
4. The cell was rocked continuously until the internal fluid ceased to flow, indicating complete hydrate formation.

(ii) MH Dissociation Test

1. Approximately 1 g of the fluidizer was injected into the rocking cell using a plunger pump from Cell No.1 to No. 5. The mass of the injected fluidizer was determined by measuring the mass of the fluidizer bottle before and after injection.
2. The flow state was documented by recording video at 10-minute intervals. The waiting time between successive injections varied between 30 minutes and 2 hours, depending on the degree of observable change.
3. Steps 1 and 2 were repeated until complete hydrate dissociation was achieved or until the gas phase volume inside the cell was depleted, preventing further injection of the fluidizer.

In our experiments, the injection interval was not predetermined but adjusted so that, after each injection, the system was allowed to evolve until no further observable changes occurred. This approach was taken in order to capture the dissociation and morphological state as close as possible to a quasi-steady condition for each injection step. This design also reflects practical considerations: in actual gas production systems for subsea methane hydrates, the degree of mixing and the time scale of blockage development cannot be precisely predicted. In severe plugging events, the only countermeasure would be to inject a large amount of strong THI. In less severe cases, however, it would be more desirable to allow sufficient time for the injected inhibitor to interact with the hydrate, thereby minimizing environmental impact by reducing the total amount of chemical injection. For these reasons, the experimental protocol was deliberately designed to vary the waiting time between injections depending on the observed system response.

Although videos were recorded at 10-minute intervals simultaneously for all five cells, the timing of fluidizer injection differed among the cells. Specifically, Cell No. 1 was injected first, while Cell No. 5 was injected last, resulting in a time lag of approximately 10 to 15 minutes between them. Consequently, in each injection sequence, Cell No. 1 represents the condition with the longest elapsed time after fluidizer injection, followed by Cells No. 2, 3, 4, and 5 in that order. As a result, the visual assessment of MH dissociation based on the sequential photographs places Cell No. 5 at a disadvantage. Nevertheless, even when accounting for this temporal discrepancy, the superiority of Cells No. 4 and No. 5 in terms of rapid MH dissociation was consistently observed across all runs because of dense fluidizer components.

Parameters

Full descriptions of used experimental parameters are given in Table S1 in Supporting Information. Table 2 summarizes the composition of the fluidizers used in this experiment. These conditions are denoted as "Set" in the table. In this study, the effects of surfactants at different concentrations were examined by injecting the fluidizers and observing the hydrate formation and dissociation behavior. In this table, regarding the composition of the fluidizer, urea, which acts as a thermodynamic hydrate inhibitor (THI), induces MH dissociation in proportion to the amount injected. Therefore, its concentration is expressed as a mass fraction relative to the total mass of the fluidizer. In contrast, additives are intended to play a

supplementary role in promoting fluidity and mixing, rather than directly contributing to MH dissociation, and are primarily assumed to interact with water. Accordingly, their concentrations are expressed based on a two-component system consisting of the additive and water, calculated as the mass of the additive divided by the combined mass of the additive and water in the fluidizer.

Fig. 2 shows the present conditions for the MH dissociation tests on the phase diagram. The present pressure and temperature conditions, i.e., 10 MPa and 283.2 K, is on the phase equilibrium curve of MH inhibited by 10 mass% urea solution. Table 3 shows the simulated concentration of the fluidizer components in the aqueous solution in the cells on the assumption of ideal complete mixing of fluidizer and the initially injected water. Based on this calculation, at the 3rd injection urea concentration reaches over 10 mass% of urea. Therefore, after infinite waiting time MH in the cell may completely dissociate. If not in the case, it means that the mixing in the cell is not enough, and MH is kinetically preserved in the system which does not reach an equilibrium state. This table further shows simulated surfactant concentrations in each cell, which estimate foaming potential at each injection stage based on CMC given in Table 2. Foaming, generation and stabilization of gas bubbles in the liquid phase, often occurs due to methane gas release during hydrate dissociation.

	No.1	No.2	No.3	No.4	No.5
No. of injection<br>Injected amount<br>Elapsed time after last injection	Urea: 30.0%<br>SDS: 0ppm	Urea: 30.0%<br>SDS: 57.1ppm	Urea: 30.0%<br>SDS: 486ppm	Urea: 30.0%<br>SDS: 1080ppm	Urea: 29.9%<br>SDS: 5200ppm
Before MH Formation					
0th<br>0g<br>0m					
1st<br>1.05g<br>26m					
2nd<br>2.04g<br>34m					
3rd<br>3.12g<br>35m					
4th<br>4.11g<br>31m					
5th<br>4.97g<br>1h30m					
6th<br>6.08g<br>45m					
7th<br>7.02g<br>36m					
8th<br>7.99g<br>4h02m					

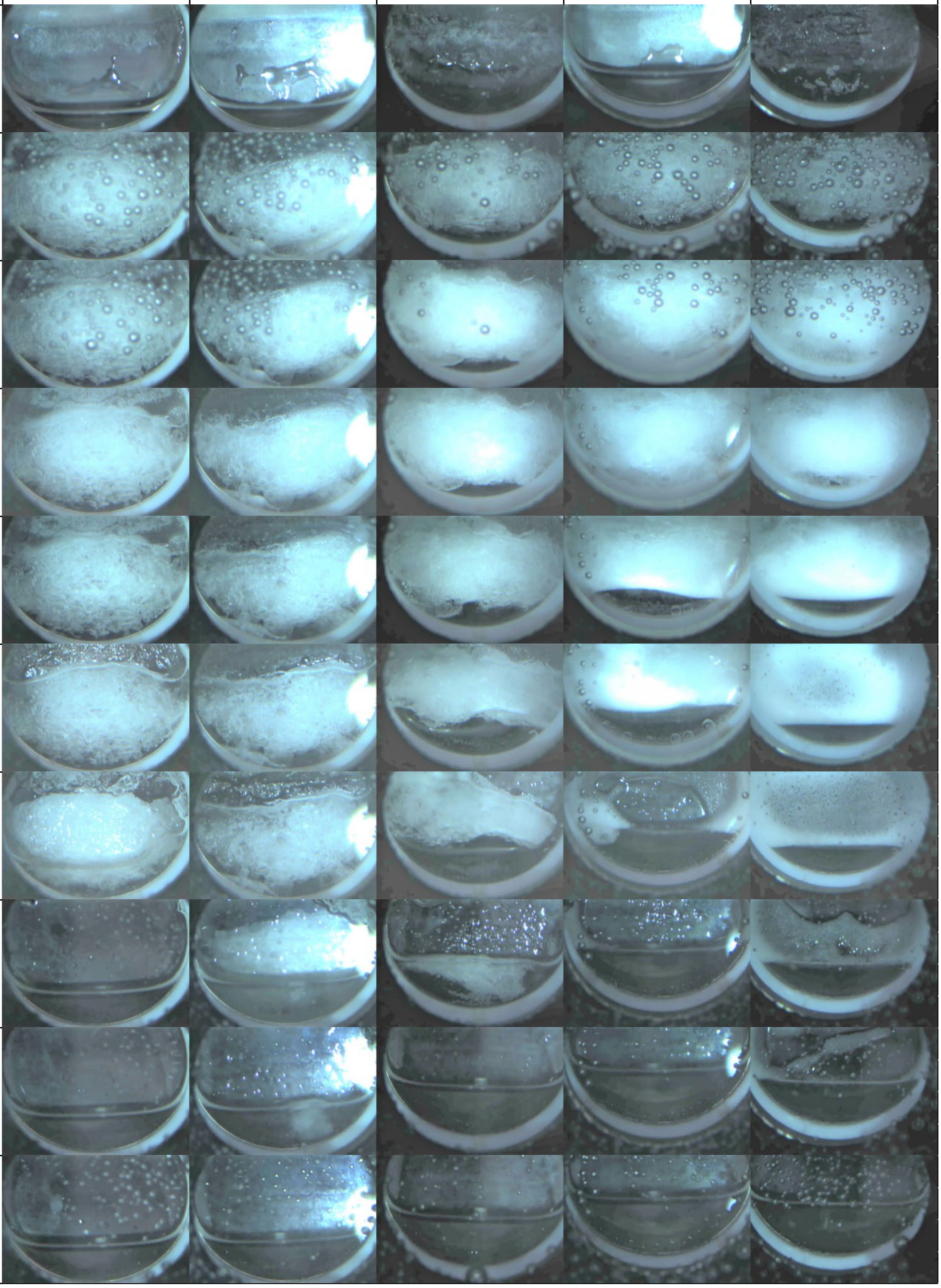

(a) set-1

Figure S1. Dissociation behavior of methane hydrates in the 10 Sets tested in this study.

	No.1	No.2	No.3	No.4	No.5
No. of injection<br>Injected amount<br>Elapsed time after last injection	Urea: 30.0 %<br>PVP: 0 %	Urea: 29.8 %<br>PVP: 0.991 %	Urea: 29.6 %<br>PVP: 1.96 %	Urea: 29.0 %<br>PVP: 4.77 %	Urea: 28.0 %<br>PVP: 9.13 %
Before MH Formation	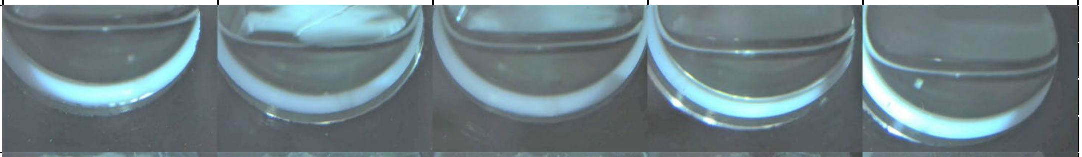				
0th<br>0g<br>0m	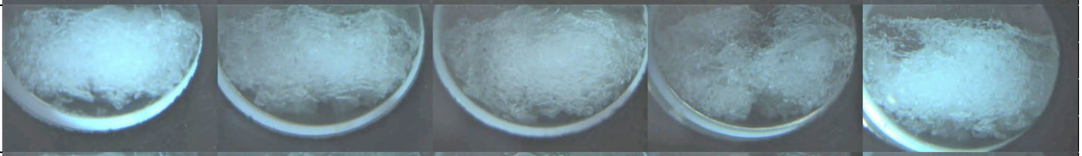				
1st<br>1.08g<br>38m	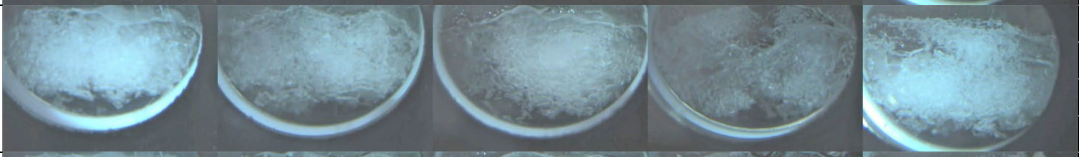				
2nd<br>2.04g<br>37m	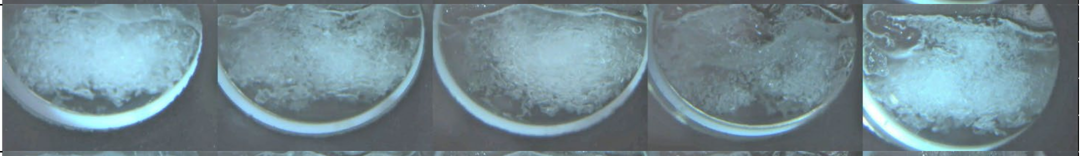				
3rd<br>2.97g<br>1h29m	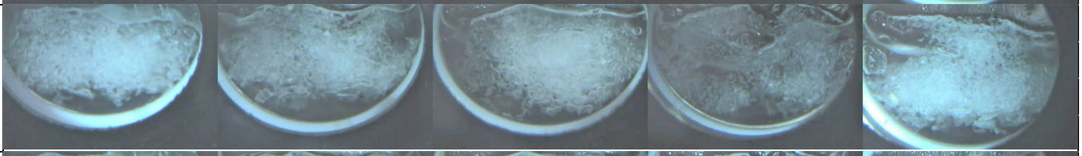				
4th<br>4.05g<br>50m	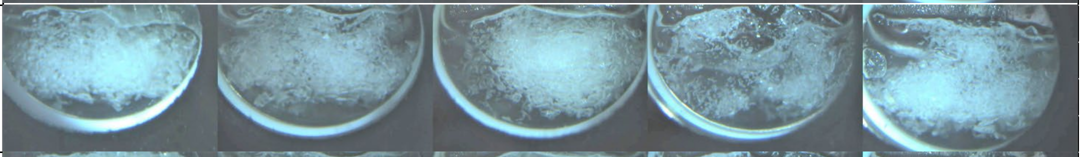				
5th<br>5.09g<br>40m	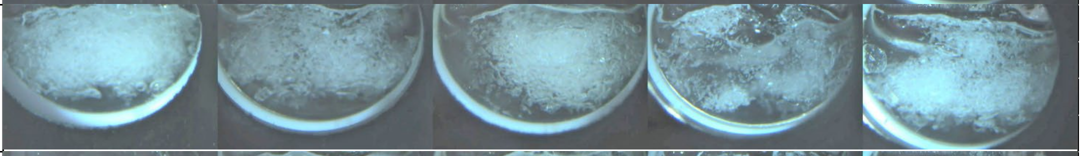				
6th<br>6.03g<br>36m	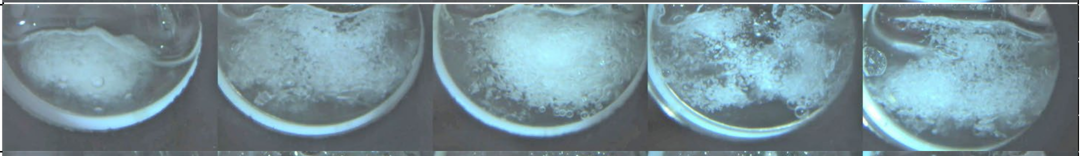				
7th<br>7.10g<br>42m	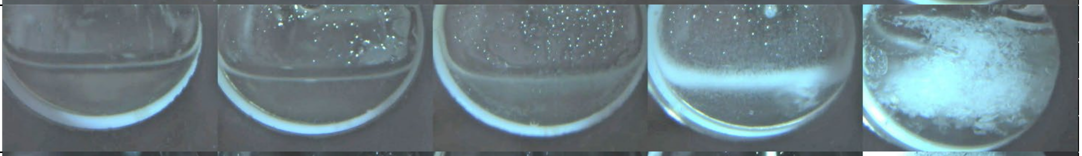				
8th<br>8.06g<br>3h01m	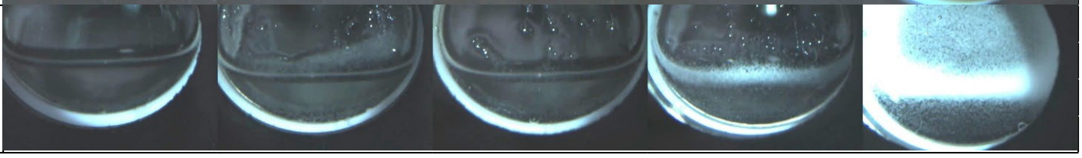				

(b) set-2

(Continue) Figure S1. Dissociation behavior of methane hydrates in the 10 Sets tested in this study.

	No.1	No.2	No.3	No.4	No.5
No. of injection<br>Injected amount<br>Elapsed time after last injection	Urea: 30.0 %<br>SO: 0 ppm	Urea: 30.0 %<br>SO: 50.0 ppm	Urea: 30.0 %<br>SO: 500ppm	Urea: 30.0 %<br>SO: 1000 ppm	Urea: 29.9 %<br>SO: 5000 ppm
Before MH formation					
0th<br>0g<br>0m					
1st<br>1.04g<br>40m					
2nd<br>2.00g<br>40m					
3rd<br>3.02g<br>41m					
4th<br>3.98g<br>1h42m					
5th<br>4.95g<br>49m					
6th<br>6.02g<br>50m					
7th<br>6.99g<br>3h05m					

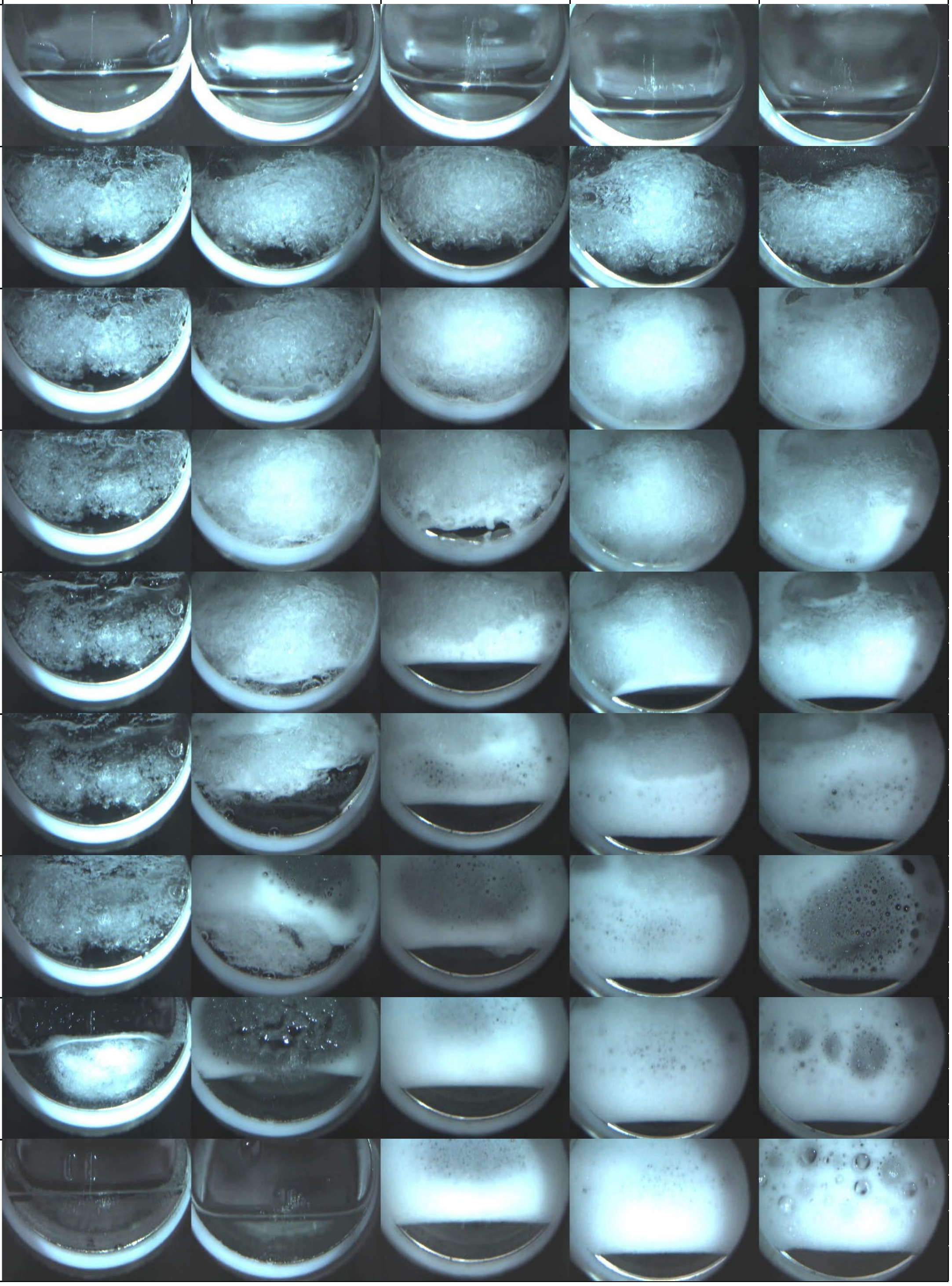

(c) set-3

(Continue) Figure S1. Dissociation behavior of methane hydrates in the 10 Sets tested in this study.

	No.1	No.2	No.3	No.4	No.5
No. of injection<br>Injected amount<br>Elapsed time after last injection	Urea: 30.0%<br>DTMAC: 0ppm	Urea: 30.0%<br>DTMAC: 40.8ppm	Urea: 30.0%<br>DTMAC: 470ppm	Urea: 30.0%<br>DTMAC: 897ppm	Urea: 29.9%<br>DTMAC: 5020ppm
Before MH formation					
0th<br>0g<br>0m					
1st<br>0.97g<br>30m					
2nd<br>2.00g<br>34m					
3rd<br>3.03g<br>41m					
4th<br>3.98g<br>1h58m					
5th<br>5.03g<br>1h21m					
6th<br>6.01g<br>1h42m					

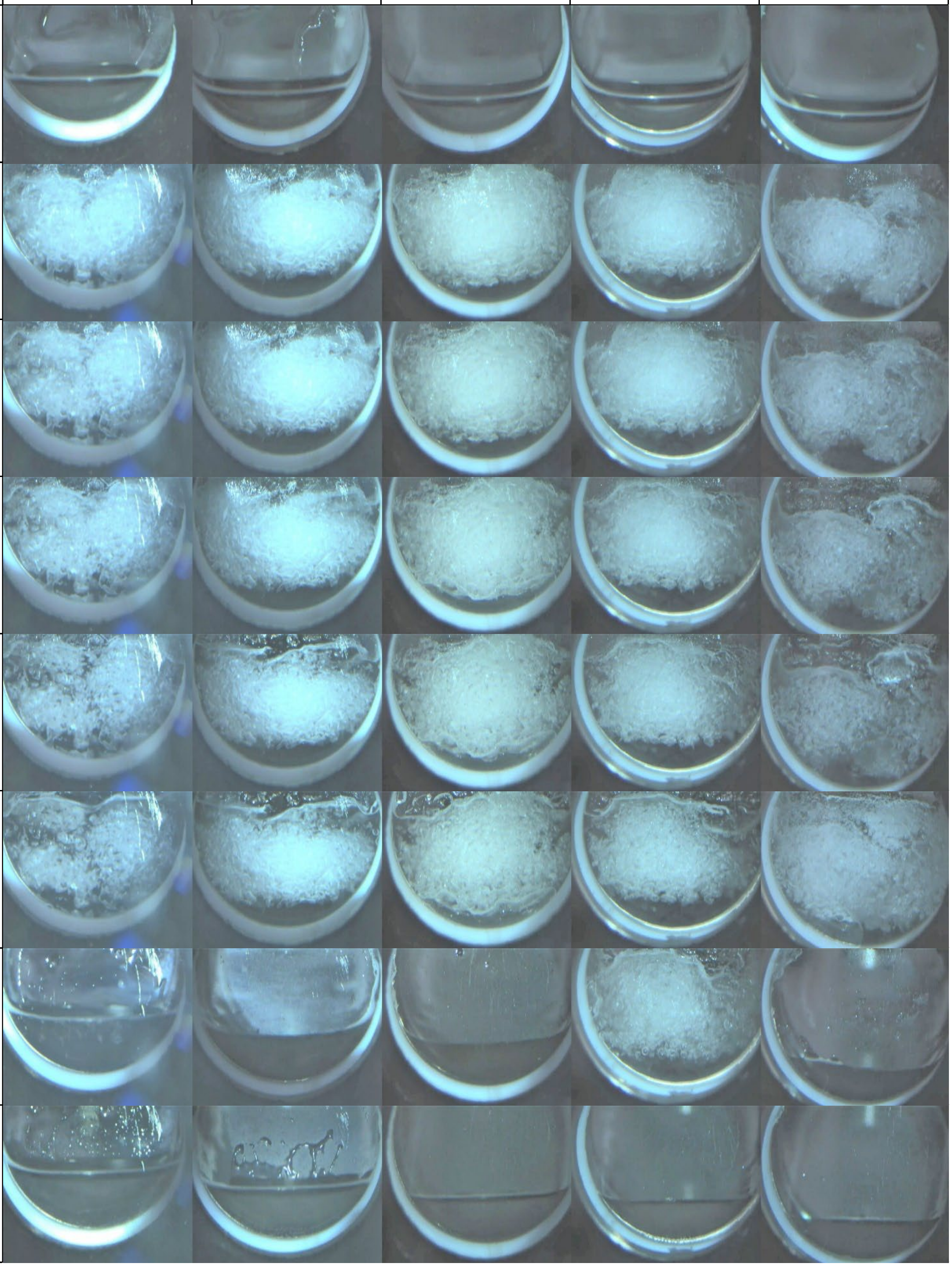

(d) set-4

(Continue) Figure S1. Dissociation behavior of methane hydrates in the 10 Sets tested in this study.

	No.1	No.2	No.3	No.4	No.5
No. of injection<br>Injected amount<br>Elapsed time after last injection	Urea: 30.0 %<br>Tween80: 0 ppm	Urea: 30.0 %<br>Tween80: 111 ppm	Urea: 30.0 %<br>Tween80: 495 ppm	Urea: 30.0 %<br>Tween80: 977 ppm	Ure29.9: %<br>Tween80: 5070 ppm
Before MH formation					
0th<br>0g<br>0m					
1st<br>1.16g<br>39m					
2nd<br>1.98g<br>30m					
3rd<br>3.07g<br>45m					
4th<br>3.97g<br>1h42m					
5th<br>4.97g<br>1h06m					
6th<br>6.13g<br>1h33m					
7th<br>6.97g<br>40m					

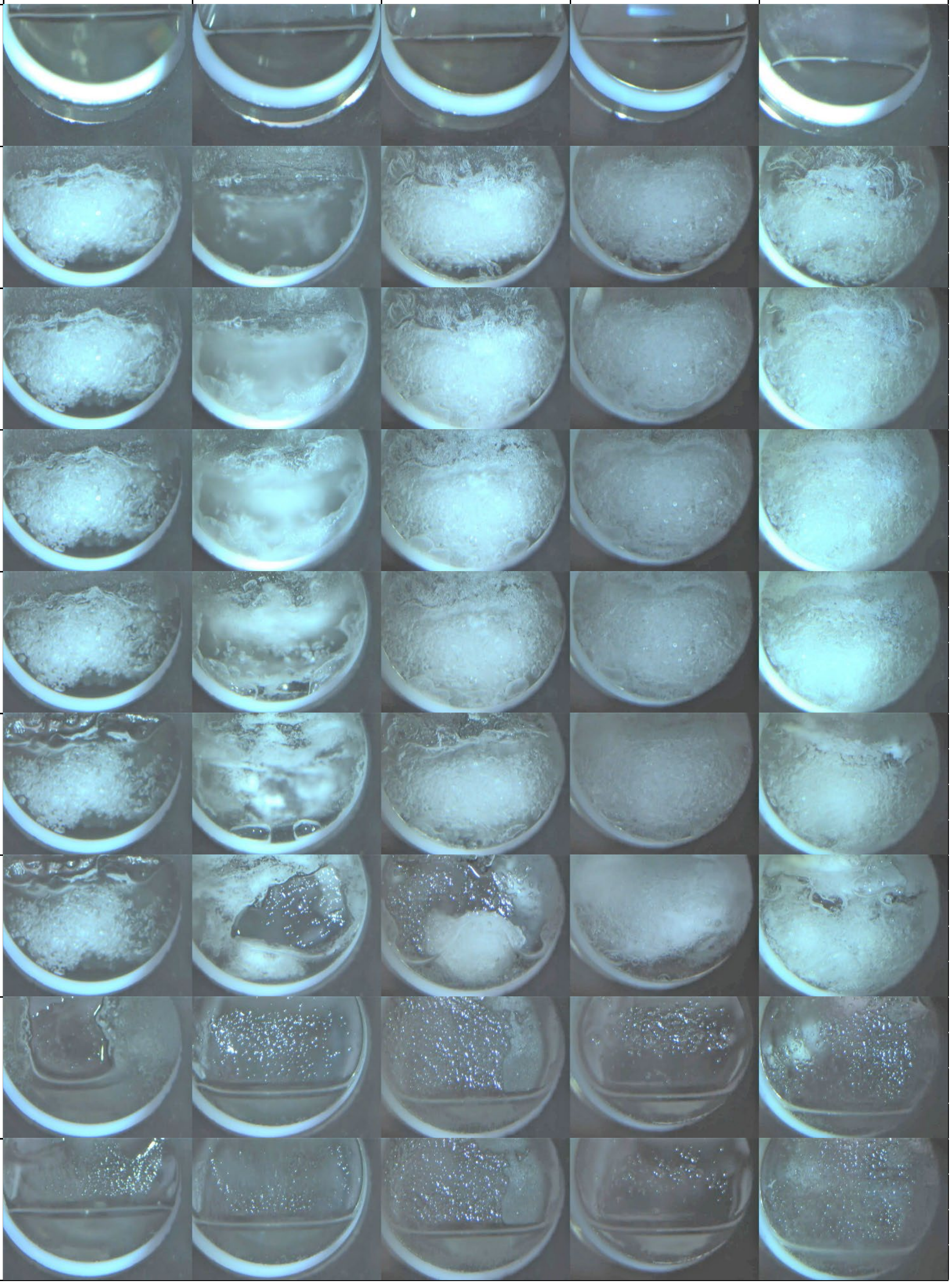

(e) set-5

(Continue) Figure S1. Dissociation behavior of methane hydrates in the 10 Sets tested in this study.

	No.1	No.2	No.3	No.4	No.5
No. of injection<br>Injected amount<br>Elapsed time after last injection	Urea: 30.0 %<br>DDBSA: 0 ppm	Urea: 30.0 %<br>DDBSA: 50.0 ppm	Urea: 30.0 %<br>DDBSA: 500 ppm	Urea: 30.0 %<br>DDBSA: 1000 p0m	Urea: 29.9 %<br>DDBSA: 5010 ppm
Before MH formation					
0th<br>0g<br>0m					
1st<br>1.01g<br>40m					
2nd<br>2.04g<br>30m					
3rd<br>3.00g<br>38m					
4th<br>4.05g<br>40m					
5th<br>4.99g<br>1h19m					
6th<br>6.03g<br>39m					
7th<br>7.01g<br>1h33m					

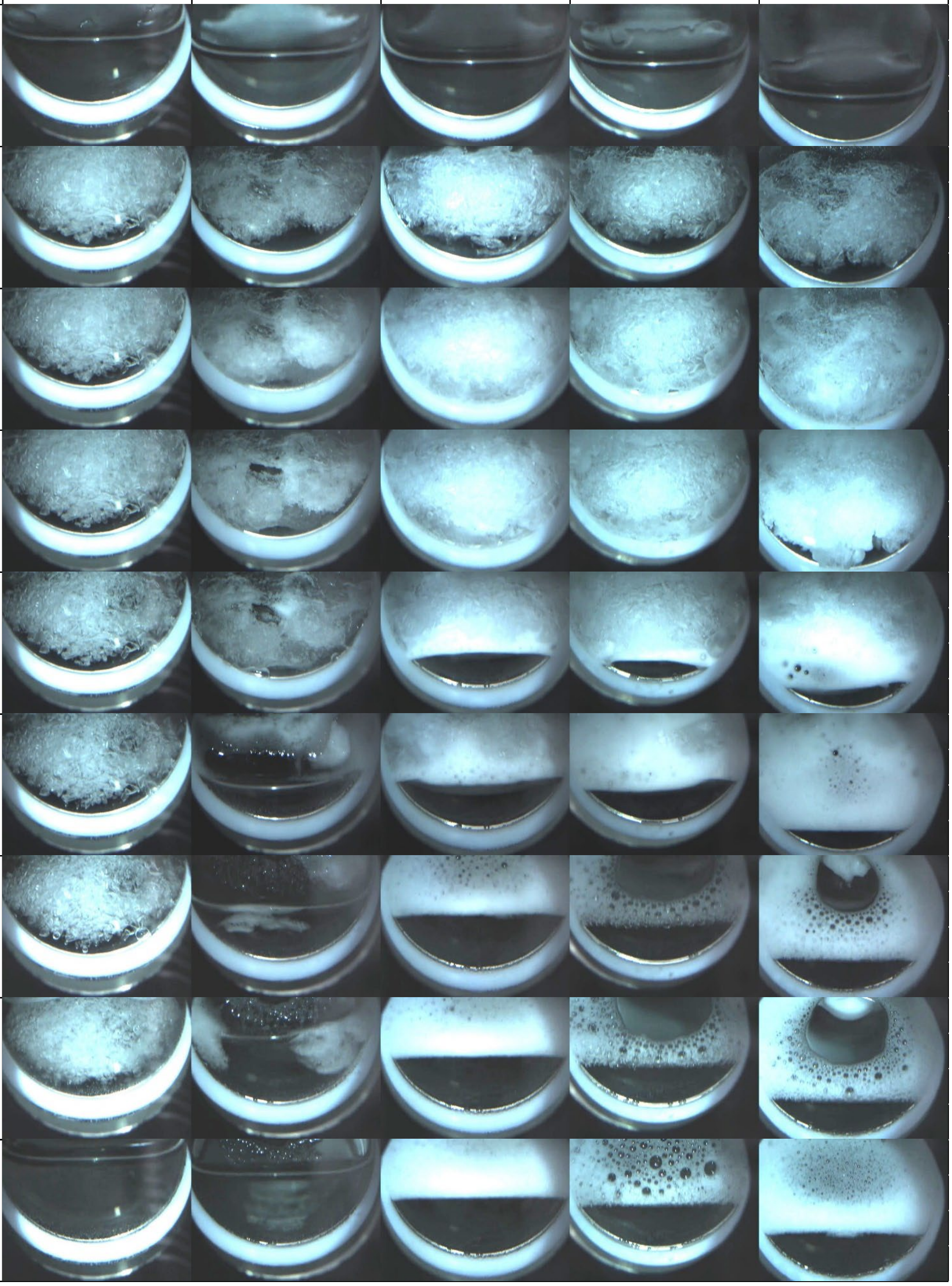

(f) set-6

(Continue) Figure S1. Dissociation behavior of methane hydrates in the 10 Sets tested in this study.

	No.1	No.2	No.3	No.4	No.5
No. of injection<br>Injected amount<br>Elapsed time after last injection	Urea: 30.0 %<br>LDAAA: 0 ppm	Urea: 30.0 %<br>LDAAA: 17.5 ppm	Urea: 30.0 %<br>LDAAA: 501 ppm	Urea: 30.0 %<br>LDAAA: 998 ppm	Urea: 29.9 %<br>LDAAA: 4950 ppm
Before MH formation	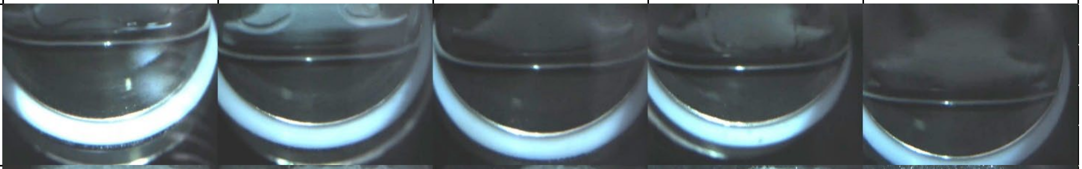				
0th<br>0g<br>0m	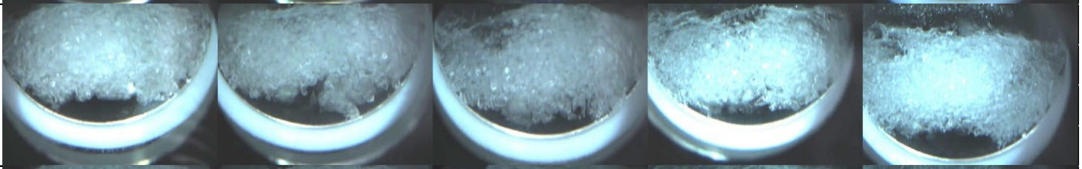				
1st<br>1.05g<br>41m	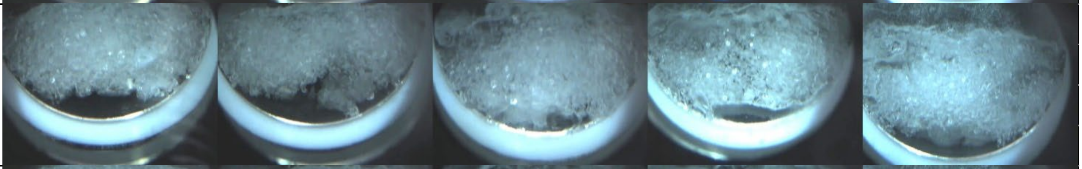				
2nd<br>2.00g<br>40m	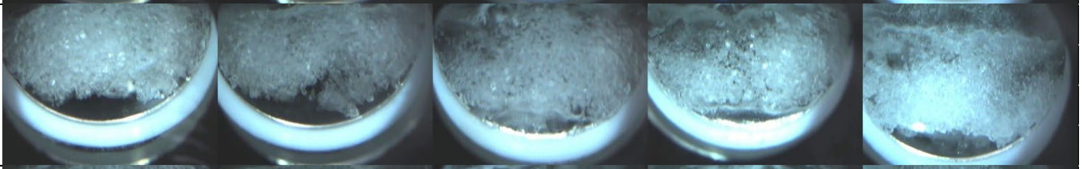				
3rd<br>3.03g<br>41m	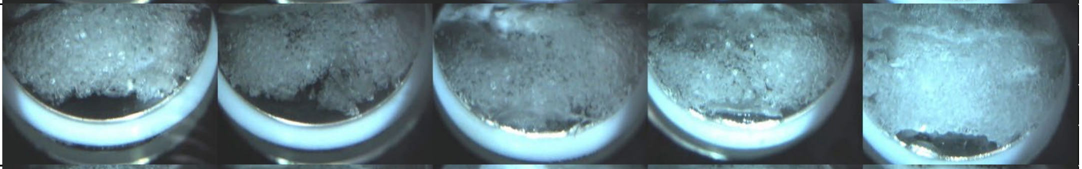				
4th<br>4.00g<br>1h21m	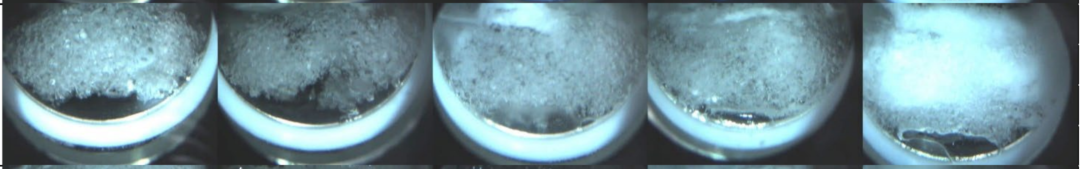				
5th<br>5.06g<br>1h01m	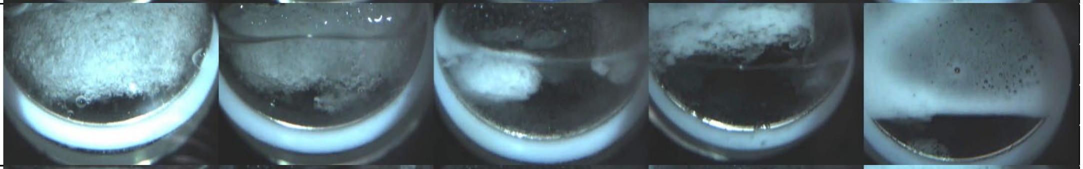				
6th<br>6.05g<br>40m	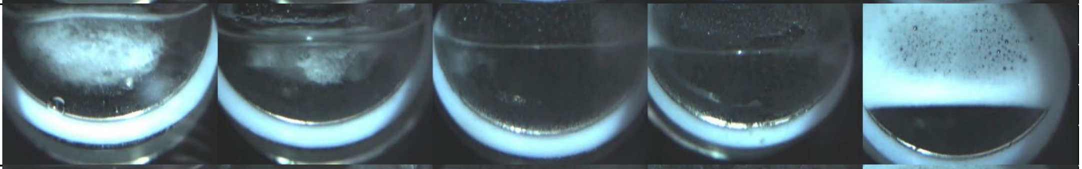				
7th<br>6.99g<br>2h33m	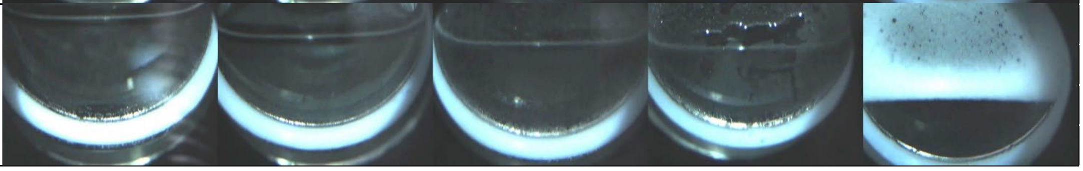				

(g) set-7

(Continue) Figure S1. Dissociation behavior of methane hydrates in the 10 Sets tested in this study.

	No.1	No.2	No.3	No.4	No.5
No. of injection<br>Injected amount<br>Elapsed time after last injection	Urea: 30.0 %<br>Saponin: 0ppm	Urea: 30.0 %<br>Saponin: 50.0ppm	Urea: 30.0 %<br>Saponin: 494ppm	Urea: 30.0 %<br>Saponin: 1000ppm	Urea: 30.0 %<br>Saponin: 5010ppm
Before MH formation					
0th<br>0g<br>0m					
1st<br>50m<br>1.00g					
2nd<br>40m<br>2.03g					
3rd<br>51m<br>3.01g					
4th<br>1h21m<br>4.00g					
5th<br>1h12m<br>5.00g					
6th<br>6.02g<br>1h01m					
7th<br>7.04g<br>2h55m					

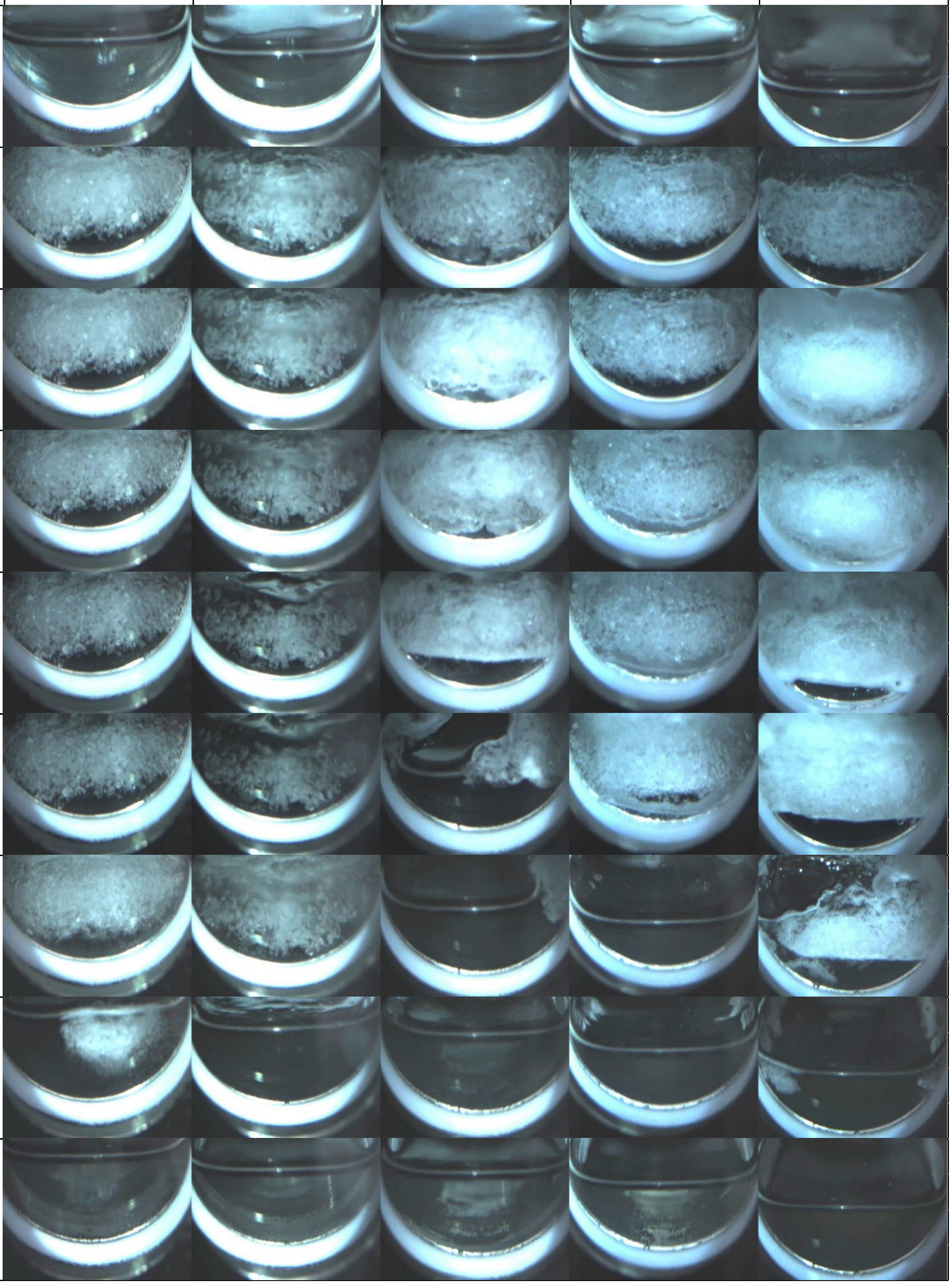

(h) set-8

(Continue) Figure S1. Dissociation behavior of methane hydrates in the 10 Sets tested in this study.

	No.1	No.2	No.3	No.4	No.5
No. of injection<br>Injected amount<br>Elapsed time after last injection	Urea: 30.0%<br>SDS: 1020ppm<br>PVP: 0%	Urea: 29.8%<br>SDS: 1010ppm<br>PVP: 0.994%	Urea: 29.6%<br>SDS: 1000ppm<br>PVP: 2.06%	Urea: 29.0%<br>SDS: 982ppm<br>PVP: 4.79%	Urea: 27.8%<br>SDS: 943ppm<br>PVP: 10.0%
Before MH Formation					
0th<br>0g<br>0m					
1st<br>0.92g<br>38m					
2nd<br>2.05g<br>40m					
3rd<br>2.95g<br>50m					
4th<br>4.14g<br>1h37m					
5th<br>4.96g<br>1h22m					
6th<br>6.02g<br>54m					
7th<br>6.93g<br>2h51m					

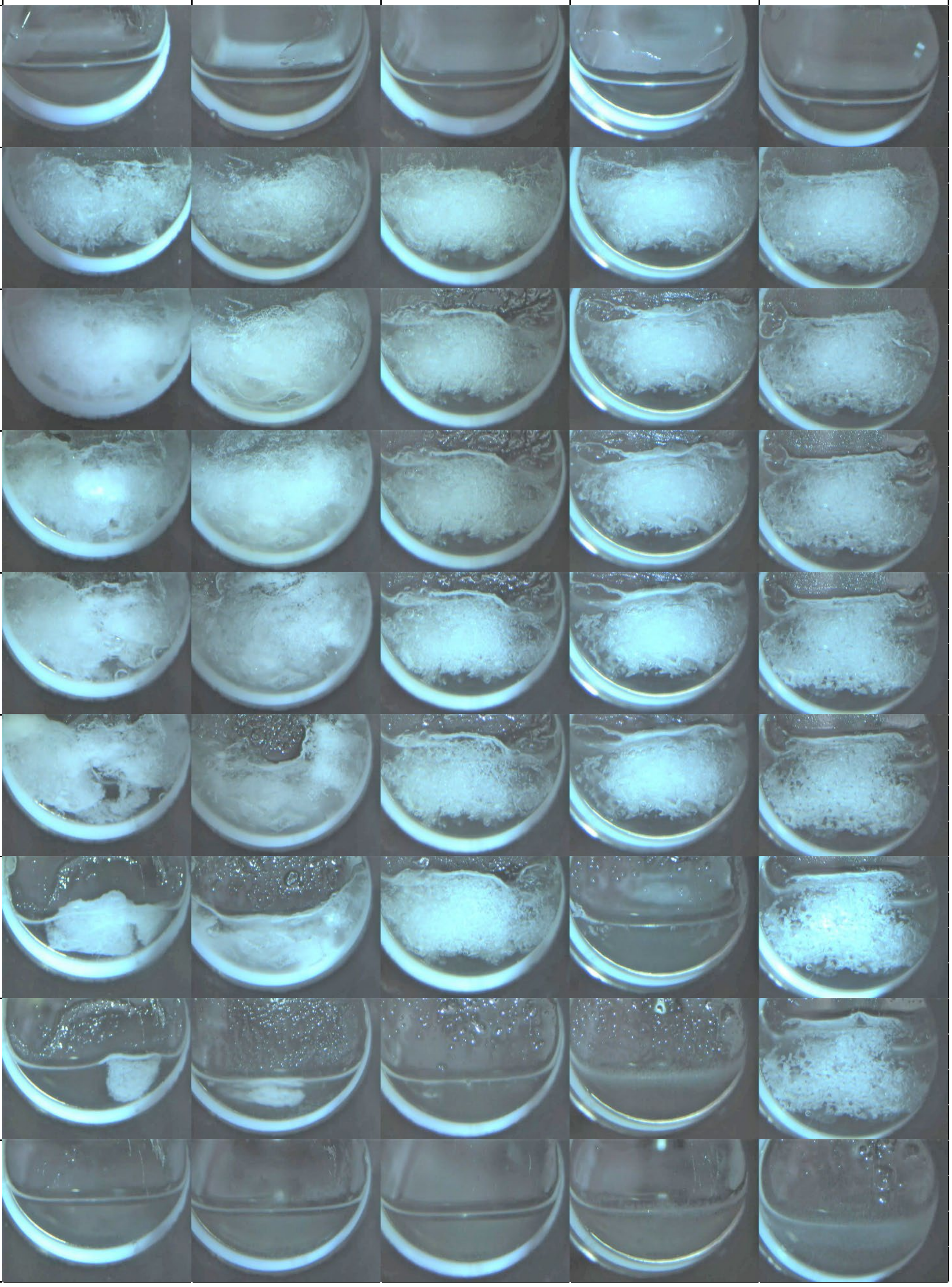

(i) set-9

(Continue) Figure S1. Dissociation behavior of methane hydrates in the 10 Sets tested in this study.

	No.1	No.2	No.3	No.4	No.5
No. of injection<br>Injected amount<br>Elapsed time after last injection	Urea: 30.0 %<br>SDS: 993 ppm<br>Tween80: 0 ppm	Urea: 30.0 %<br>SDS: 992 ppm<br>Tween80: 102 ppm	Urea: 30.0 %<br>SDS: 992 ppm<br>Tween80: 502 ppm	Urea: 30.0 %<br>SDS: 992 ppm<br>Tween80: 1010 ppm	Urea: 29.9 %<br>SDS: 989 ppm<br>Tween80: 5070 ppm
Before MH formation					
0th<br>0g<br>0m					
1st<br>1.06g<br>34m					
2nd<br>2.05g<br>28m					
3rd<br>3.04g<br>42m					
4th<br>4.00g<br>1h52m					
5th<br>4.95g<br>1h05m					
6th<br>6.03g<br>2h03m					
7th<br>7.02g<br>4h37m					

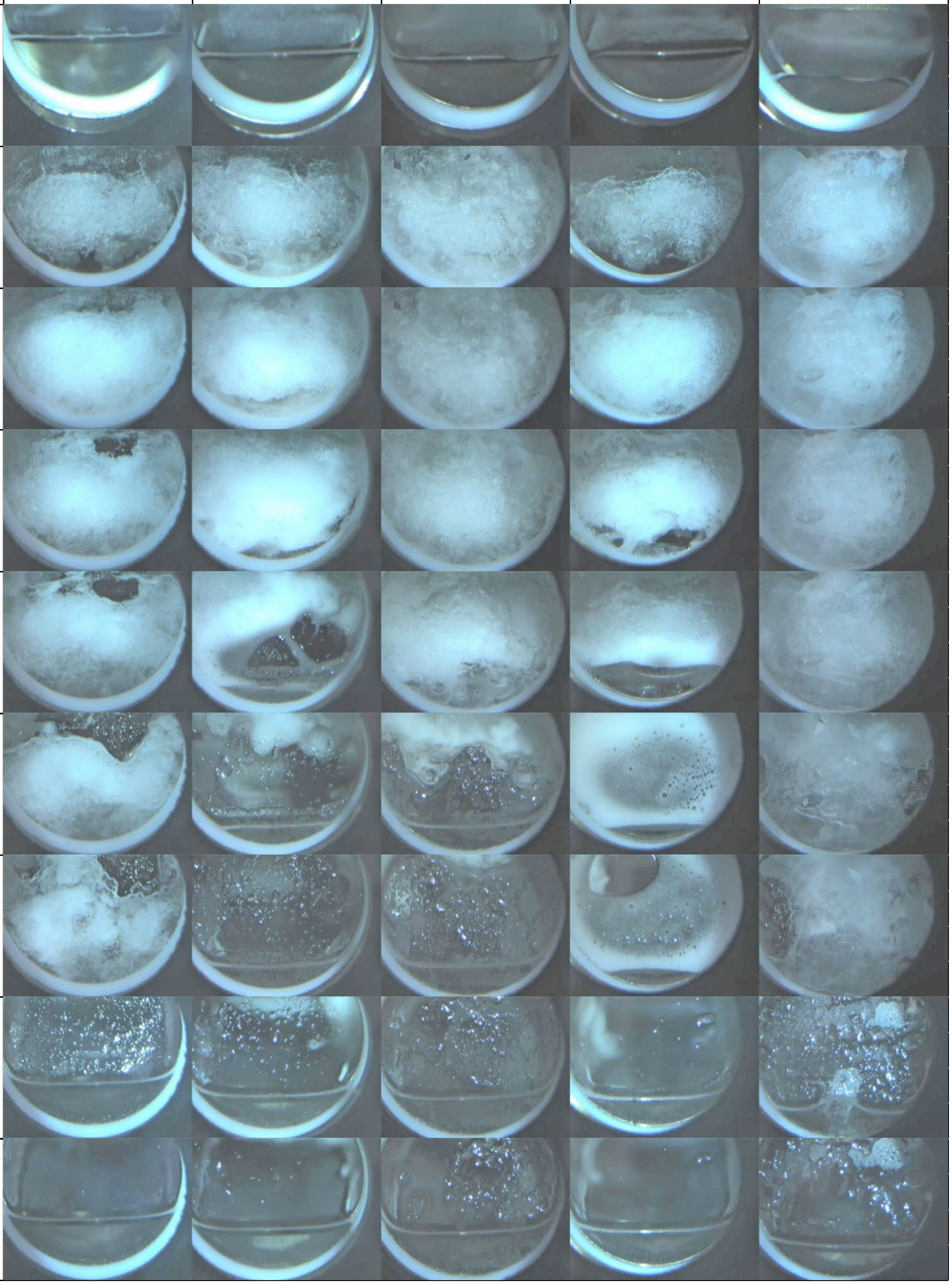

(j) set-10

(Continue) Figure S1. Dissociation behavior of methane hydrates in the 10 Sets tested in this study.

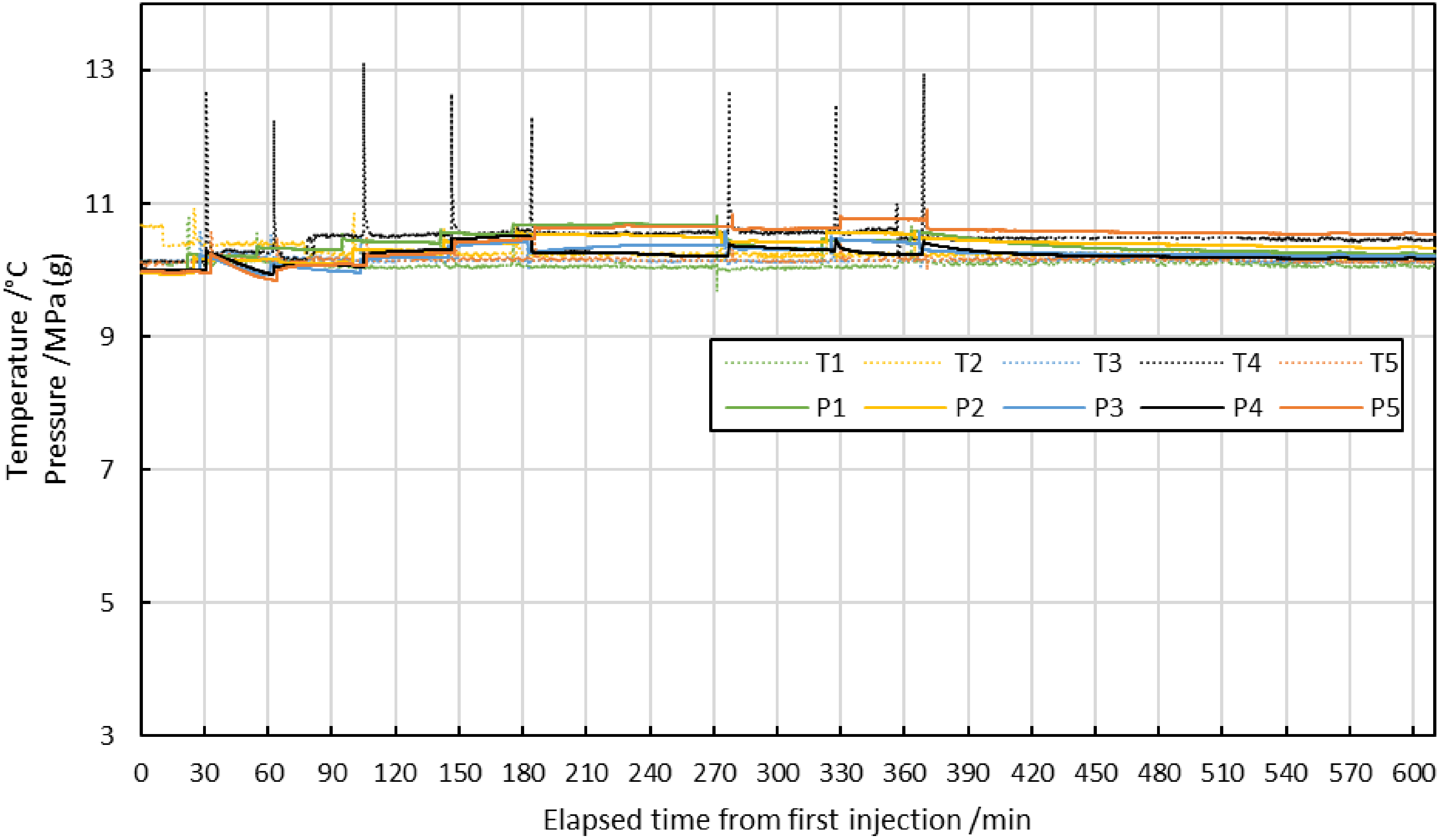

(a) set-1

Figure S2. Pressure and temperature trends during MH dissociation tests.

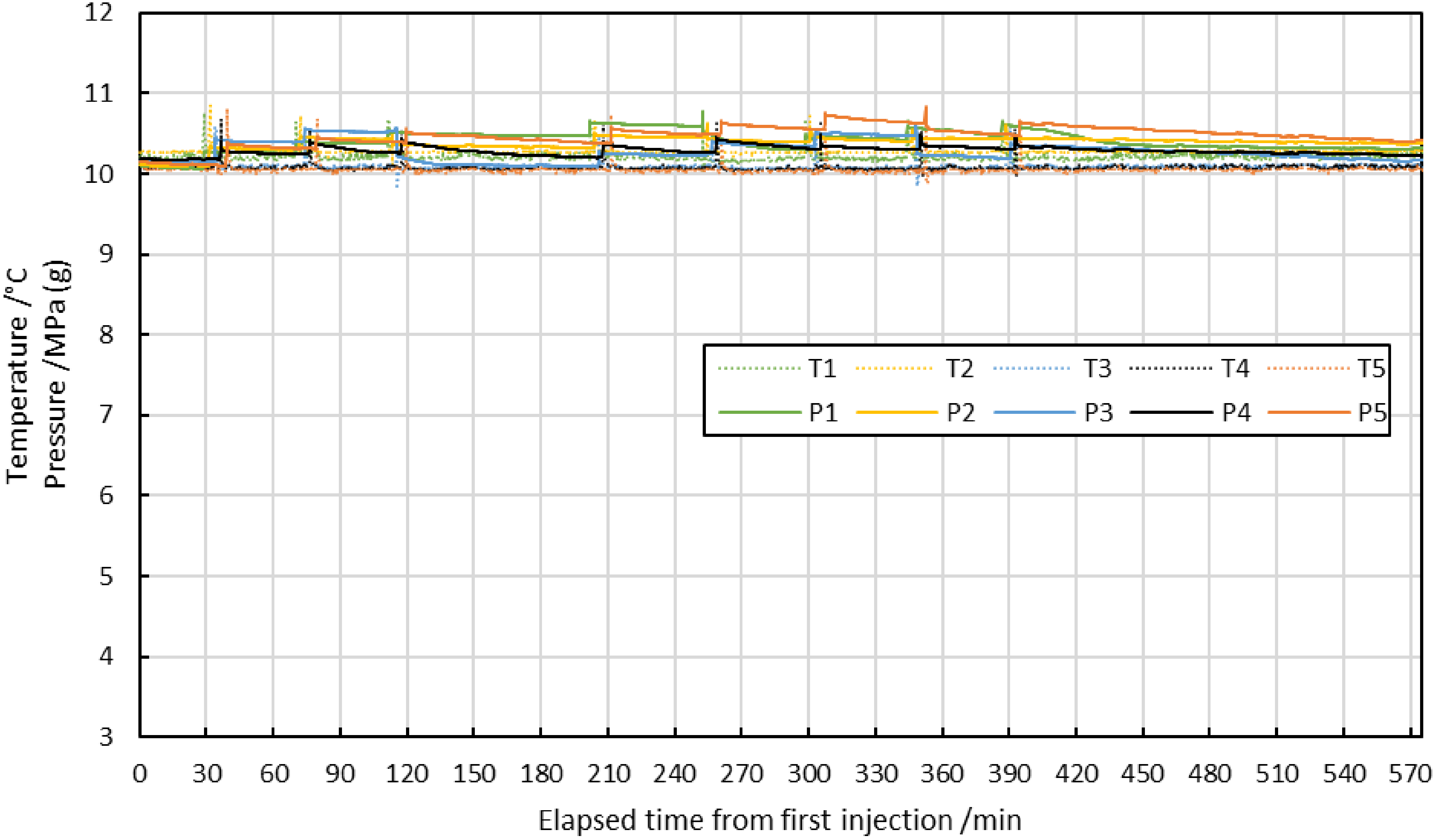

(b) set-2

(Continue) Figure S2. Pressure and temperature trends during MH dissociation tests.

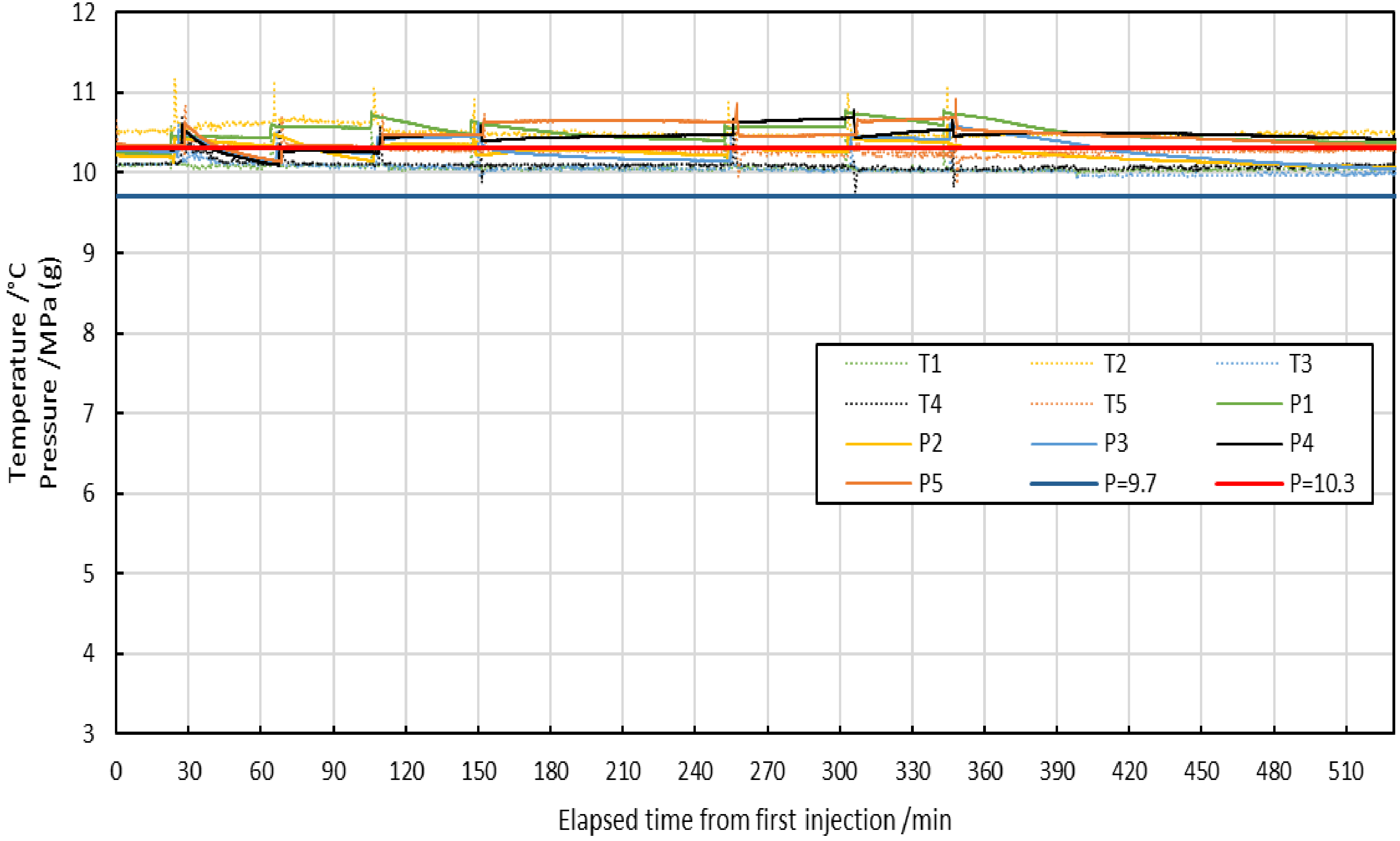

(c) set-3

(Continue) Figure S2. Pressure and temperature trends during MH dissociation tests.

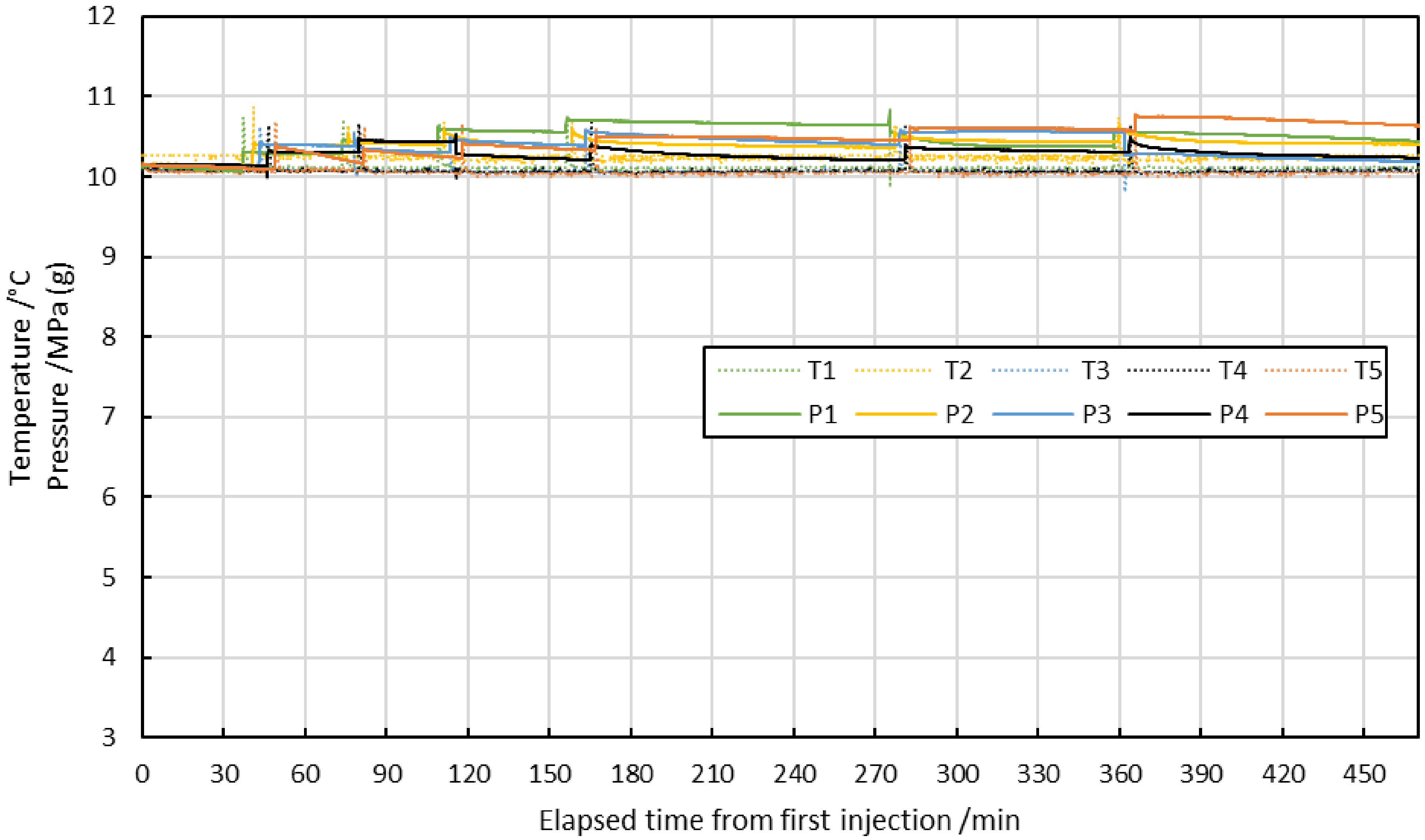

(d) set-4

(Continue) Figure S2. Pressure and temperature trends during MH dissociation tests.

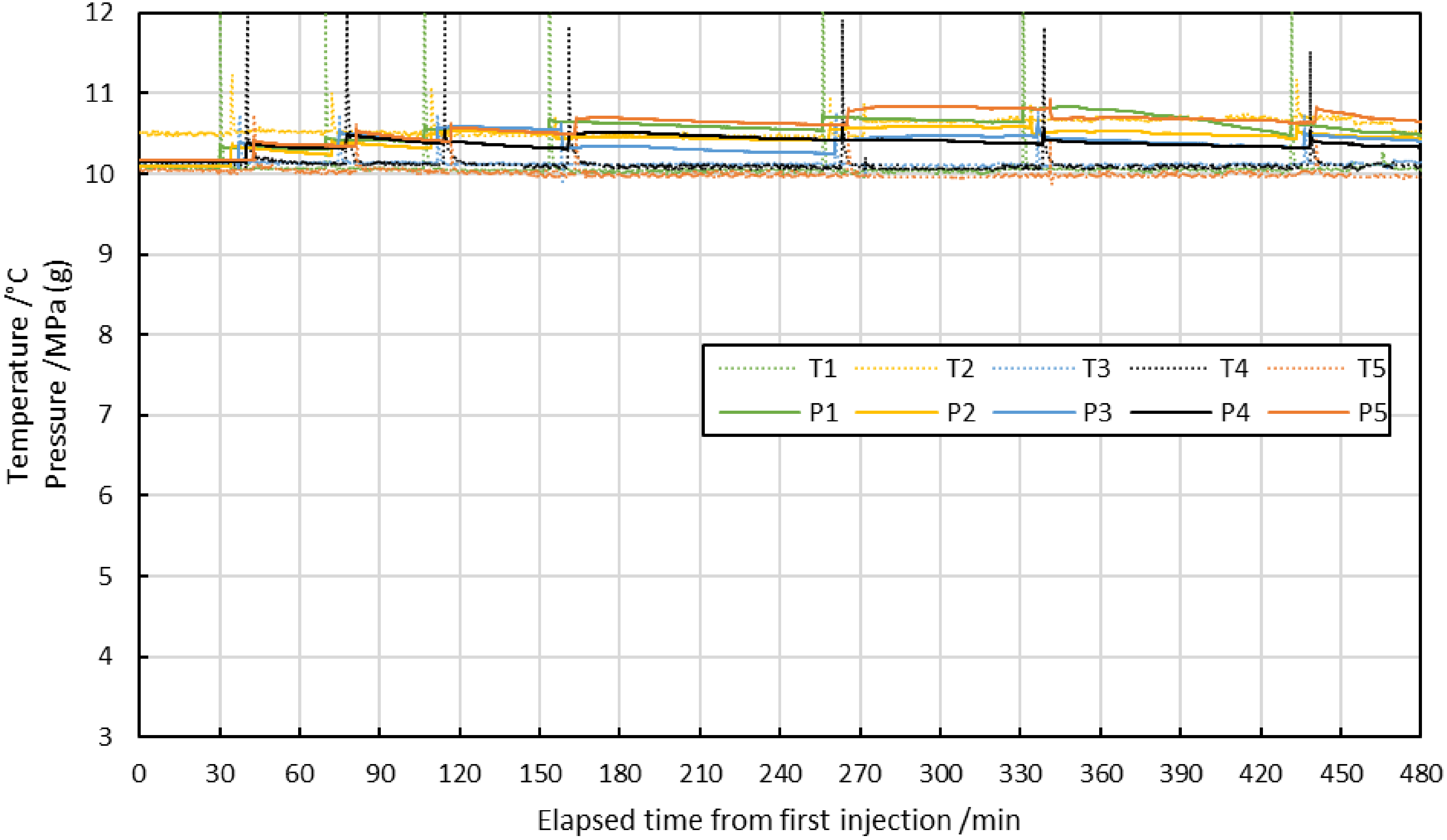

(e) set-5

(Continue) Figure S2. Pressure and temperature trends during MH dissociation tests.

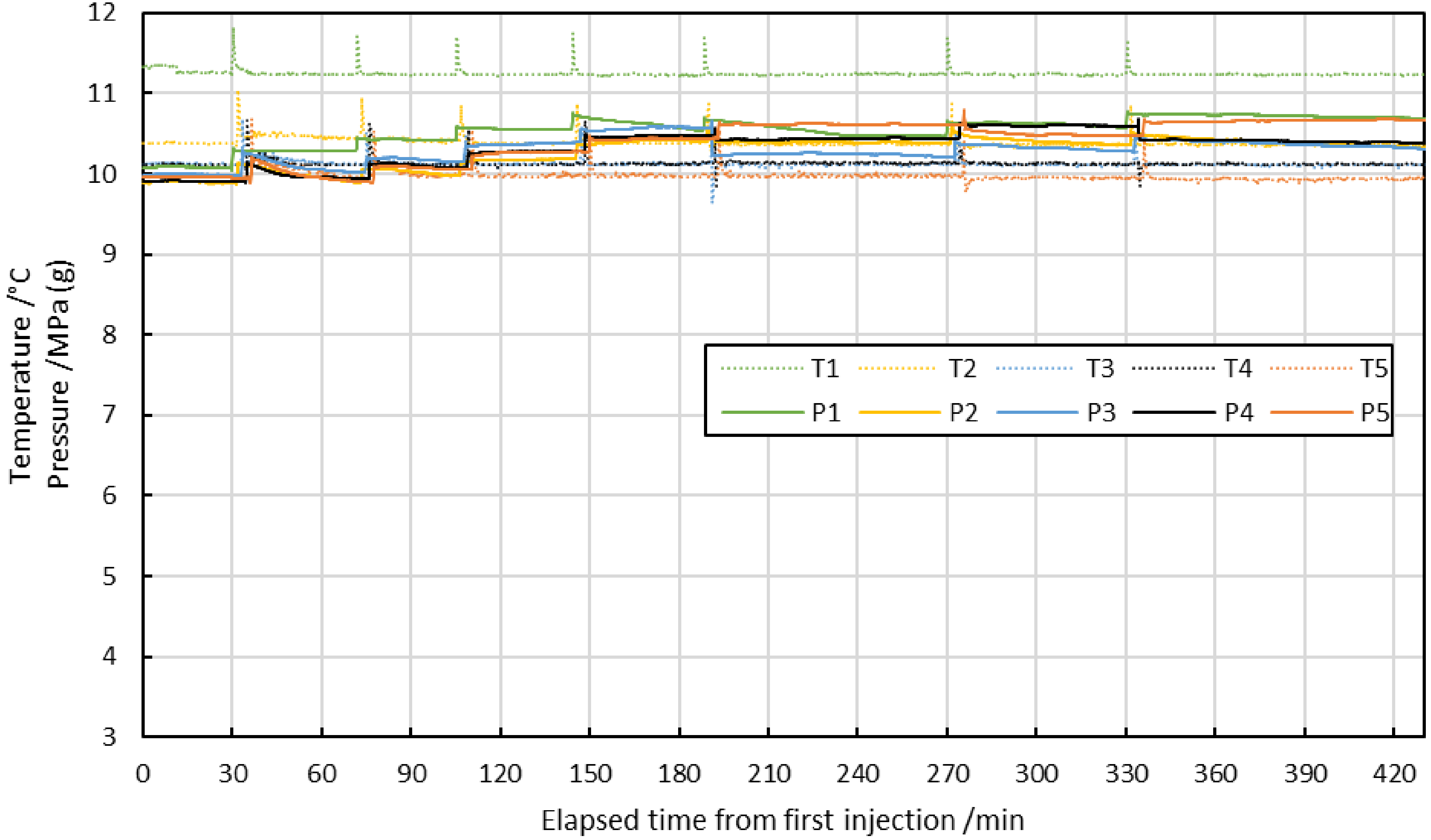

(f) set-6 (Thermometer T1 indicated shifted tempeartures.)

(Continue) Figure S2. Pressure and temperature trends during MH dissociation tests.

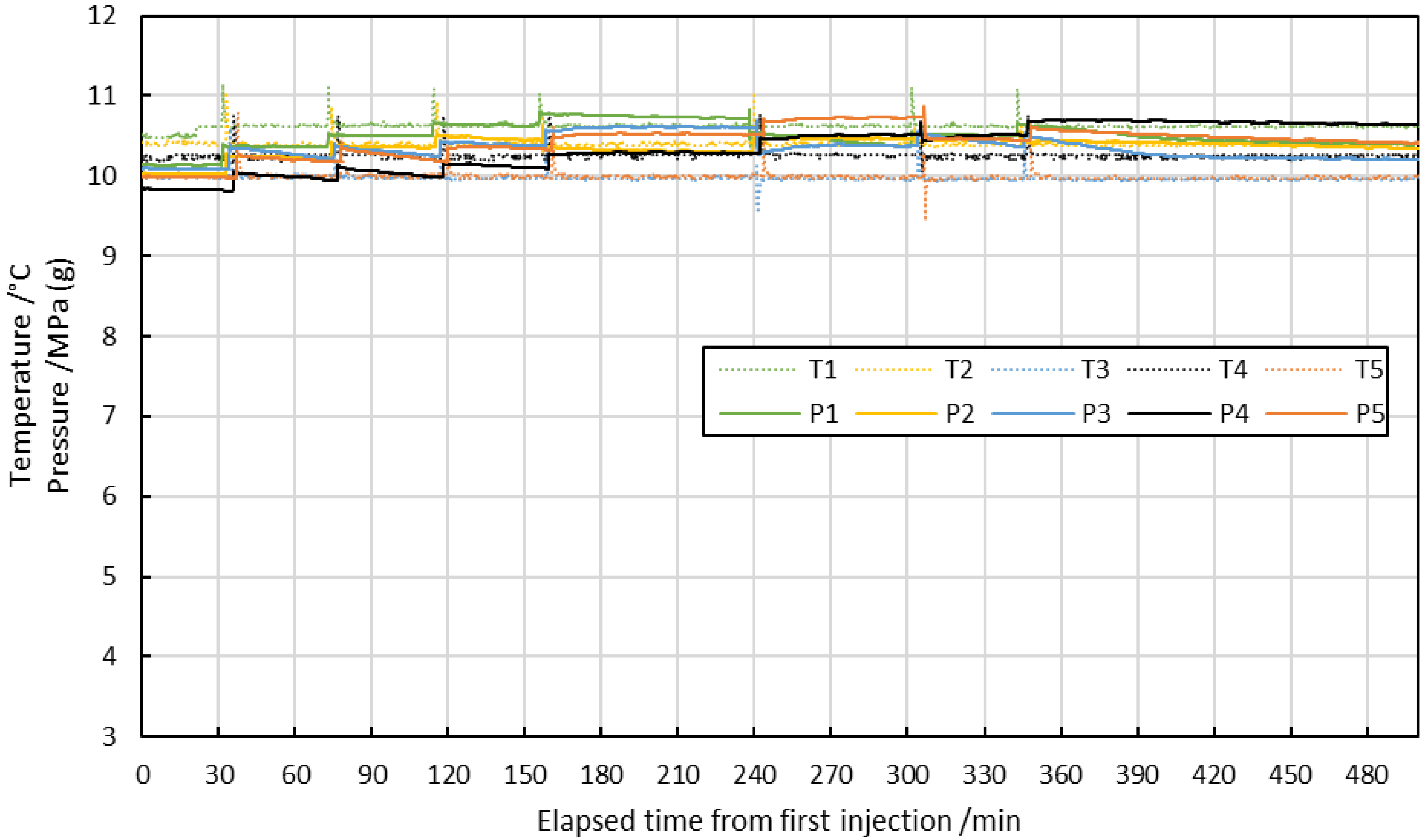

(g) set-7

(Continue) Figure S2. Pressure and temperature trends during MH dissociation tests.

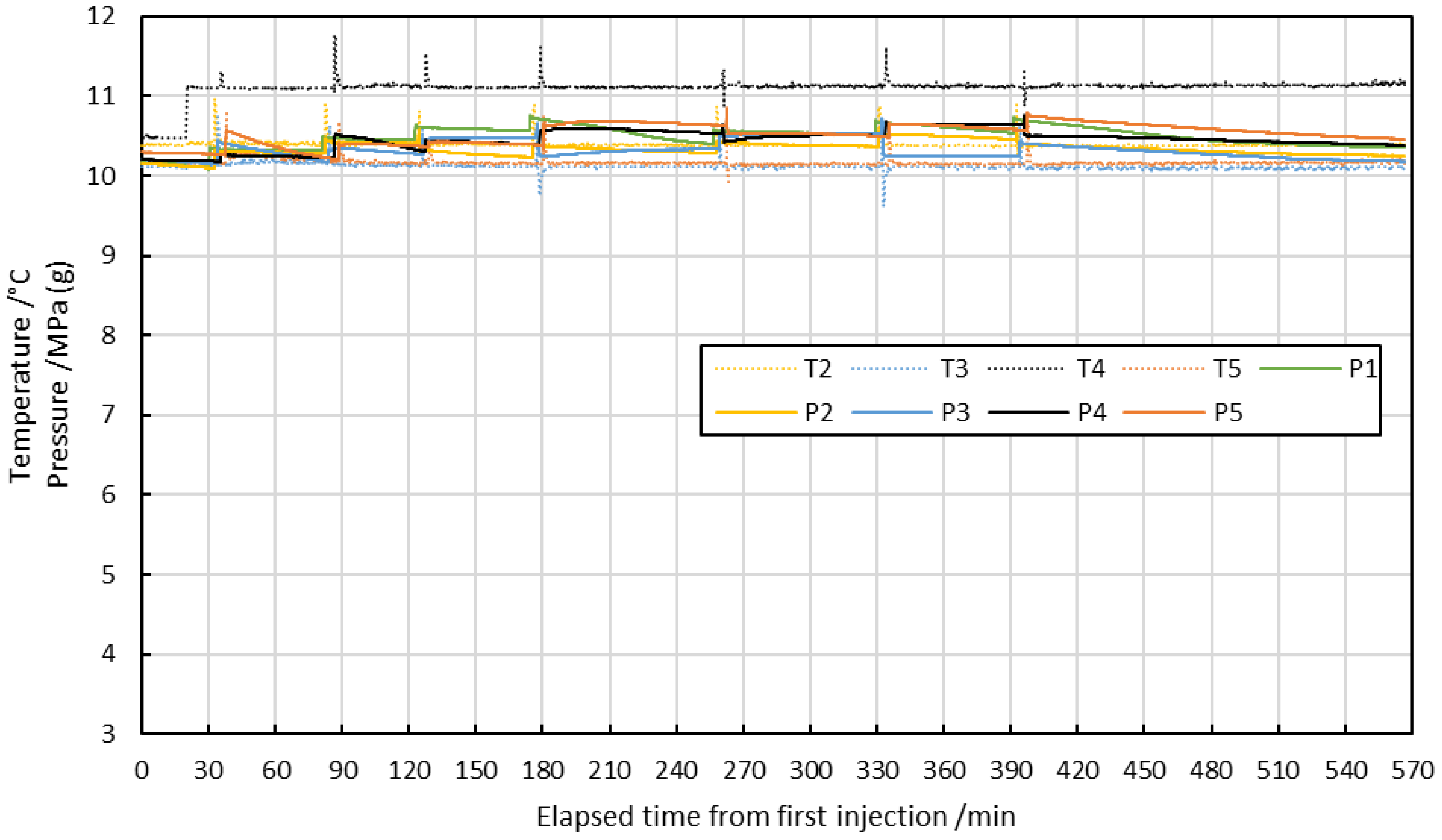

(h) set-8 (Thermometer T4 indicated shifted tempeartures.)

(Continue) Figure S2. Pressure and temperature trends during MH dissociation tests.

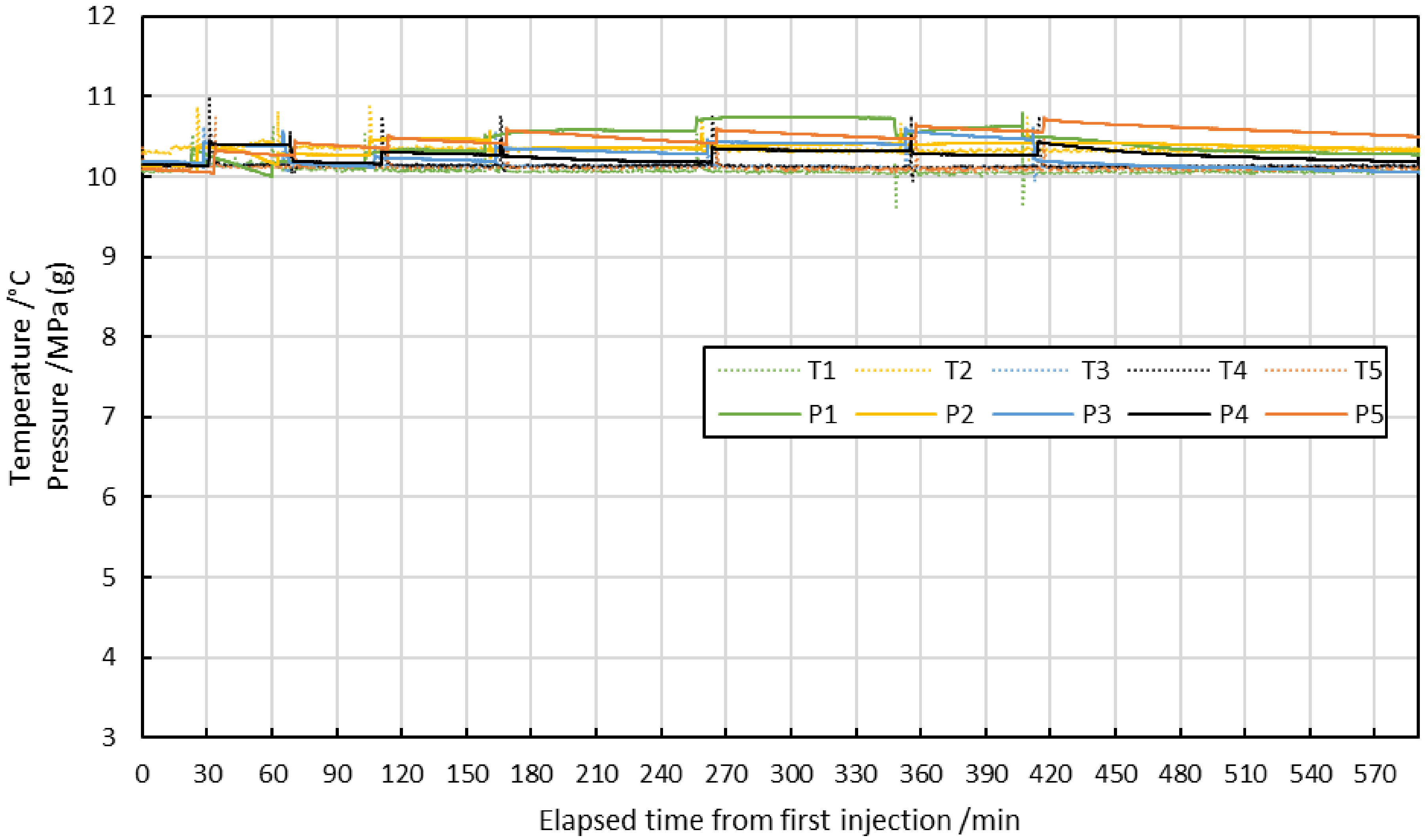

(i) set-9

(Continue) Figure S2. Pressure and temperature trends during MH dissociation tests.

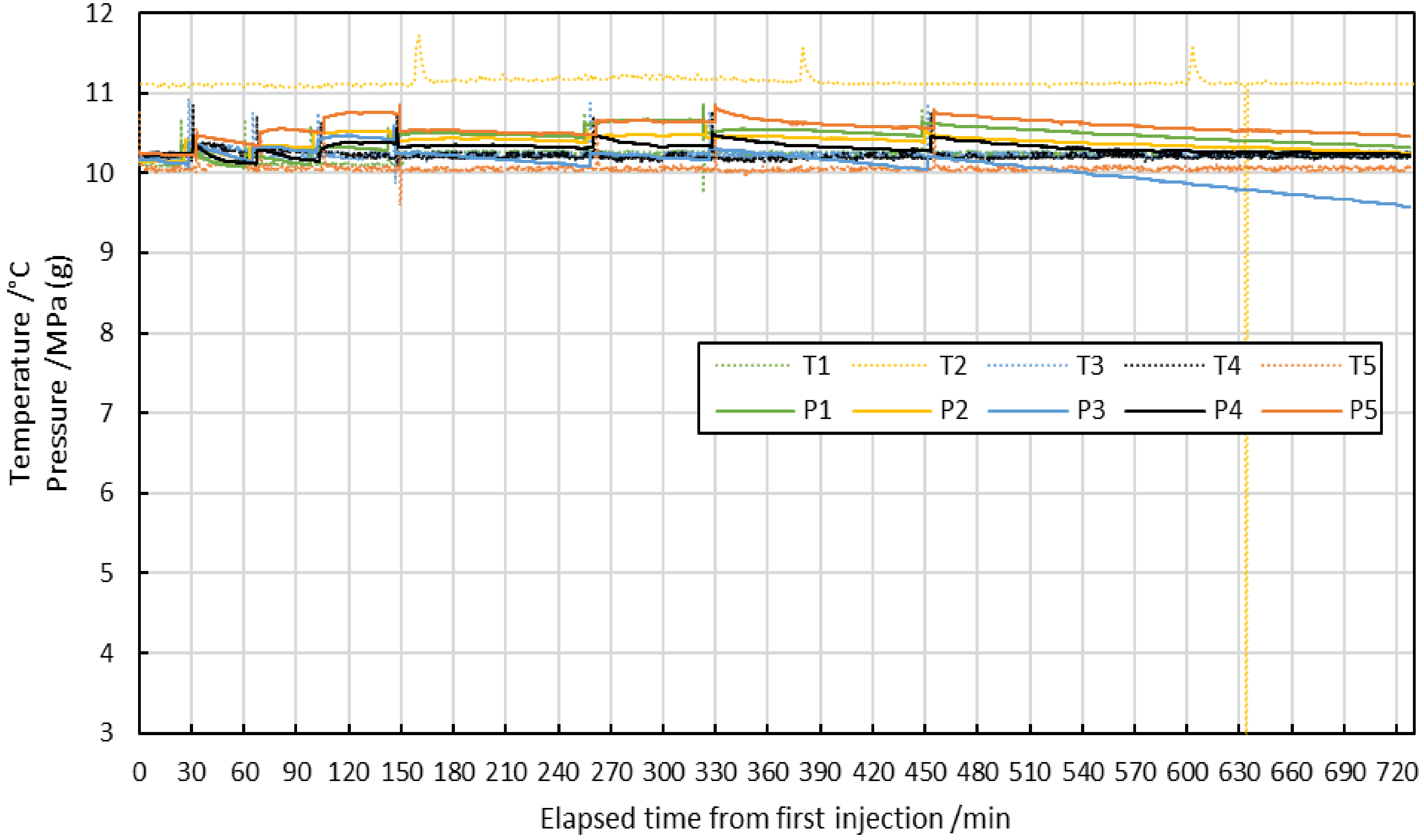

(j) set-10 (Thermometer T2 was found to have an open circuit at aroud 630 min.)

(Continue) Figure S2. Pressure and temperature trends during MH dissociation tests.

Table S1. Detail parameters of fluidizer compositions. Compositions of the components are shown as that in binary mixture of water and the component, i.e., (mass of a component)/(mass of water and a component).

Cell	Component	Set-1	Set-2	Set-3	Set-4	Set-5	Set-6	Set-7	Set-8	Set-9	Set-10
No.1	Component 1	Urea	Urea	Urea	Urea	Urea	Urea	Urea	Urea	Urea	Urea
	-composition	30.0%	30.0%	30.0%	30.0%	30.0%	30.0%	30.0%	30.0%	30.0%	30.0%
	Component 2	SDS	PVP	SO	DTMAC	Tween80	DDBSA	LDAAA	Saponin	SDS	SDS
	-composition	0	0	0	0	0	0	0	0	1016ppm	993ppm
	Component 3									PVP	Tween80
	-composition									0	0
No.2	Component 1	Urea	Urea	Urea	Urea	Urea	Urea	Urea	Urea	Urea	Urea
	-composition	30.0%	29.8%	30.0%	30.0%	30.0%	30.0%	30.0%	30.0%	29.8%	30.0%
	Component 2	SDS	PVP	SO	DTMAC	Tween80	DDBSA	LDAAA	Saponin	SDS	SDS
	-composition	57.1ppm	0.991%	50.0ppm	40.8ppm	111ppm	50.0ppm	17.5ppm	50.0ppm	1009ppm	992ppm
	Component 3									PVP	Tween80
	-composition									0.994%	102ppm
No.3	Component 1	Urea	Urea	Urea	Urea	Urea	Urea	Urea	Urea	Urea	Urea
	-composition	30.0%	29.6%	30.0%	30.0%	30.0%	30.0%	30.0%	30.0%	29.6%	30.0%
	Component 2	SDS	PVP	SO	DTMAC	Tween80	DDBSA	LDAAA	Saponin	SDS	SDS
	-composition	486ppm	1.96%	500ppm	470ppm	495ppm	500ppm	501ppm	494ppm	1001ppm	992ppm
	Component 3									PVP	Tween80
	-composition									2.06%	502ppm
No.4	Component 1	Urea	Urea	Urea	Urea	Urea	Urea	Urea	Urea	Urea	Urea
	-composition	30.0%	29.0%	30.0%	30.0%	30.0%	30.0%	30.0%	30.0%	29.0%	30.0%
	Component 2	SDS	PVP	SO	DTMAC	Tween80	DDBSA	LDAAA	Saponin	SDS	SDS
	-composition	1080ppm	4.77%	1000ppm	897ppm	977ppm	1000ppm	998ppm	1000ppm	982ppm	992ppm
	Component 3									PVP	Tween80
	-composition									4.79%	1010ppm
No.5	Component 1	Urea	Urea	Urea	Urea	Urea	Urea	Urea	Urea	Urea	Urea
	-composition	29.9%	28.0%	29.9%	29.9%	29.9%	29.9%	29.9%	29.9%	27.8%	29.9%
	Component 2	SDS	PVP	SO	DTMAC	Tween80	DDBSA	LDAAA	Saponin	SDS	SDS
	-composition	5200ppm	9.13%	5000ppm	5020ppm	5070ppm	5010ppm	4950ppm	5010ppm	943ppm	989ppm
	Component 3									PVP	Tween80
	-composition									10.0%	5070ppm
